# Molecular mechanism of specific DNA sequence recognition by NRF1

**DOI:** 10.1093/nar/gkad1162

**Published:** 2023-12-06

**Authors:** Ke Liu, Weifang Li, Yuqing Xiao, Ming Lei, Ming Zhang, Jinrong Min

**Affiliations:** Hubei Key Laboratory of Genetic Regulation and Integrative Biology, School of Life Sciences, Central China Normal University, Wuhan 430079, PR China; Hubei Key Laboratory of Genetic Regulation and Integrative Biology, School of Life Sciences, Central China Normal University, Wuhan 430079, PR China; Hubei Key Laboratory of Genetic Regulation and Integrative Biology, School of Life Sciences, Central China Normal University, Wuhan 430079, PR China; Hubei Key Laboratory of Genetic Regulation and Integrative Biology, School of Life Sciences, Central China Normal University, Wuhan 430079, PR China; Hubei Key Laboratory of Genetic Regulation and Integrative Biology, School of Life Sciences, Central China Normal University, Wuhan 430079, PR China; Hubei Key Laboratory of Genetic Regulation and Integrative Biology, School of Life Sciences, Central China Normal University, Wuhan 430079, PR China

## Abstract

Nuclear respiratory factor 1 (NRF1) regulates the expression of genes that are vital for mitochondrial biogenesis, respiration, and various other cellular processes. While NRF1 has been reported to bind specifically to GC-rich promoters as a homodimer, the precise molecular mechanism governing its recognition of target gene promoters has remained elusive. To unravel the recognition mechanism, we have determined the crystal structure of the NRF1 homodimer bound to an ATGCGCATGCGCAT dsDNA. In this complex, NRF1 utilizes a flexible linker to connect its dimerization domain (DD) and DNA binding domain (DBD). This configuration allows one NRF1 monomer to adopt a U-turn conformation, facilitating the homodimer to specifically bind to the two TGCGC motifs in the GCGCATGCGC consensus sequence from opposite directions. Strikingly, while the NRF1 DBD alone could also bind to the half-site (TGCGC) DNA of the consensus sequence, the cooperativity between DD and DBD is essential for the binding of the intact GCGCATGCGC sequence and the transcriptional activity of NRF1. Taken together, our results elucidate the molecular mechanism by which NRF1 recognizes specific DNA sequences in the promoters to regulate gene expression.

## Graphical Abstract

**Graphical Abstract:**
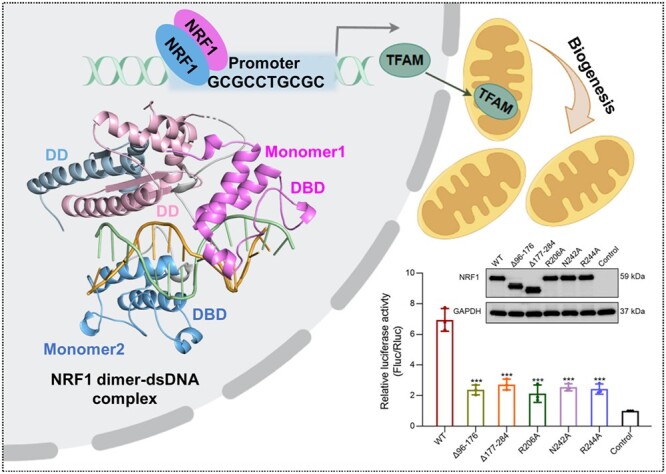

## Introduction

Mitochondria, as essential organelles, play a vital role in supplying cellular energy. Their functions and biogenesis depend on proteins not only encoded by the mitochondrial genome but also by many nuclear genes. These nuclear gene products are indispensable for various crucial processes in the mitochondria, such as transcription, translation, and replication of mitochondrial DNA (mtDNA), mitochondrial respiration and the import of other proteins into the mitochondria (1). Among these nuclear gene products, Nuclear Respiratory Factor 1 (NRF1) and NRF2 are central to maintaining the overall functionality and biogenesis of mitochondria. They have been directly linked to the expression of genes involved in mitochondrial respiration, mtDNA transcription and replication, and mitochondrial protein import (1,2). Thus, NRF1 and NRF2 ensure efficient energy production and cellular function in mitochondria.

**Figure 1.**
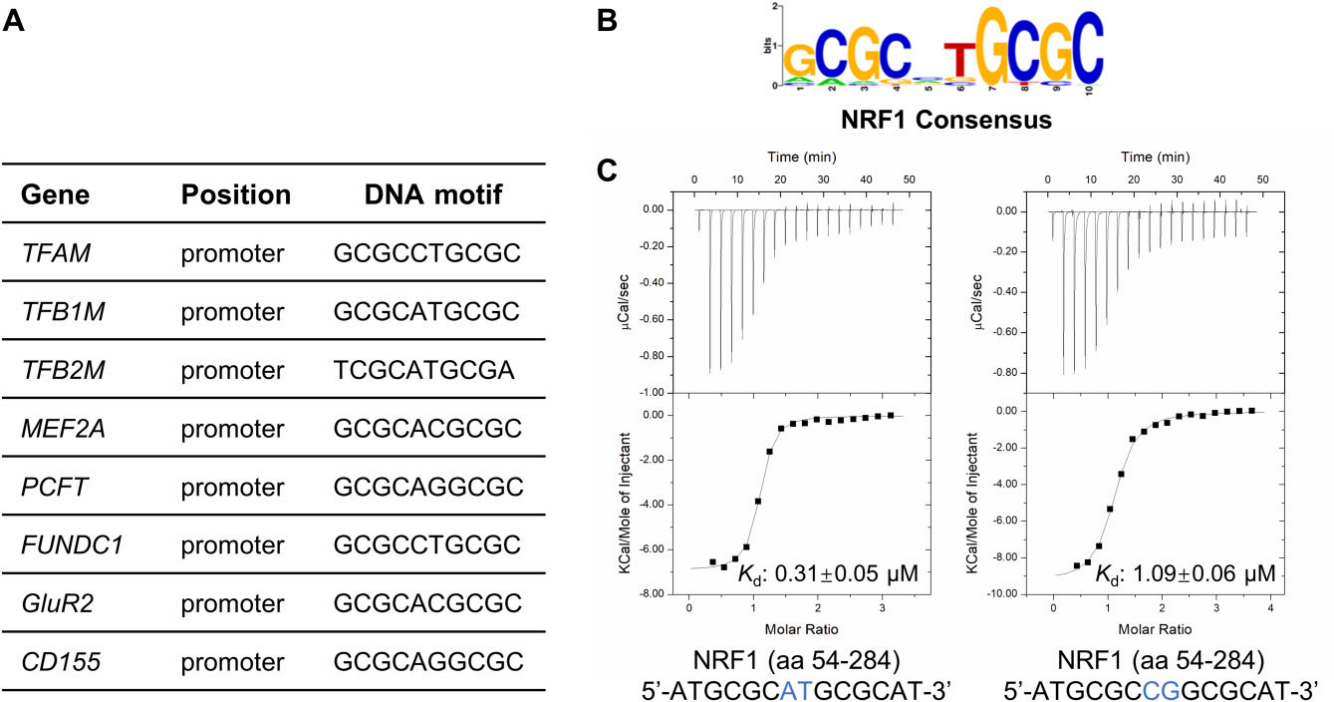
The NRF1 homodimer prefers binding to the GCGCATGCGC DNA sequence. **(A)** Reported NRF1 binding sequences in the promoters of some human genes. **(B)** Predominant DNA binding motif of the NRF1. The predominant motif is taken from https://jaspar.genereg.net/ with the ID MA0506.1. **(C)** ITC binding curves of NRF1 (aa 54–284) to ATGCGCATGCGCAT and ATGCGCCGGCGCAT DNA sequences, respectively. Only one strand of the DNA duplex is shown. The nucleotides of the central spacer between two GCGC half-sites are colored blue.

NRF1 was initially identified as a transcription activator of cytochrome c by binding to the cytochrome c promoter as a homodimer (3–5). Subsequently, a consensus sequence GCGCNTGCGC (N represents any nucleotide) was identified as the DNA binding motif of NRF1 (6), which has also been detected in the promoters of many other nuclear genes relevant to mitochondrial biogenesis and respiration, such as the promoters of reductase (ubiquinone-binding protein) complex (6), cytochrome oxidase assembly factor COX17 (7), myocyte enhancer factor 2A (MEF2A) (8), and ferridoxin reductase (FDXR) (9). Importantly, NRF1 also activates the expression of several mitochondrial transcription factors, including mitochondrial transcription factor A (TFAM), mitochondrial transcription factor B1 (TFB1M), and TFB2M (Figure 1A) (10,11). These transcription factors are required for the initiation of mtDNA transcription, mtDNA packaging (12), and dimethylation of 12S rRNA (13). Loss of NRF1 function results in partial loss of mtDNA and a peri-implantation lethal phenotype (14).

NRF1 also regulates cellular processes beyond mitochondrial biogenesis. For example, NRF1 has been found to recruit Mixed lineage leukemia 4 (MLL4) to activate the expression of Growth hormone-releasing hormone (GHRH)-neuronal genes during hypothalamus development (15). NRF1 promotes the expression of proton-coupled folate transporter (PCFT) and facilitates obligatory intestinal folate absorption (16). NRF1 also can upregulate the expression of the mitophagy receptor FUN14 domain-containing protein 1 (FUNDC1), thereby enhancing mitophagy (17). Additionally, NRF1, in conjunction with Peroxisome proliferator-activated receptor γ coactivator 1 α (PGC1α), regulates the telomere transcription and elevates the level of telomeric repeat-containing RNA (TERRA), playing a key role in maintaining telomere integrity (18). It has been reported that all of these regulatory processes are mediated by NRF1 recognizing its consensus GCGCNTGCGC motif (Figure 1A). Moreover, this consensus sequence has been found in the promoters of other genes, including the promoters the CXC chemokine receptor 4 (CXCR4) (19), the AMPA receptor subunit glutamate receptor 2 (GluR2) (20), the human poliovirus receptor CD155 (21) and others (4) (Figure 1A). Thus, NRF1 acts on a broad spectrum of target genes and exhibits a broader role in the integration of diverse cellular functions.

Although numerous studies have shown that NRF1 binds to a consensus sequence in the promoters of its target genes, the molecular mechanisms governing its recognition of this consensus sequence have not been elucidated. To this end, we performed isothermal titration calorimetry (ITC) binding experiments to analyze the DNA binding specificity of human NRF1, and determined its double-strand DNA (dsDNA) complex structures, including the fragments covering the dimerization and DNA binding tandem domain (DD-DBD) or DBD alone. Our structural, mutagenesis and luciferase reporter assay results revealed that, although the NRF1 DBD alone is capable of recognizing the TGCGC DNA motif, the cooperativity between DD and DBD is essential for the binding of the intact GCGCATGCGC sequence and the transcriptional activity of NRF1. Collectively, our findings elucidate the molecular mechanism by which NRF1 recognizes specific DNA sequences to regulate gene expression, which also paves the way for further investigations into the function of NRF1.

## Materials and methods

### Cloning, expression and purification

We designed the protein constructs based on the predicted NRF1 model by AlphaFold2 and the reported DNA binding data of NRF1 (5). Briefly, the DNA sequences encoding human NRF1 fragments, including aa 1–284, aa 54–284, aa 98–177, aa 54–177 and aa 177–284, were subcloned into the pET28-MHL vector to generate N-terminal His_6_-tagged fusion proteins, respectively. NRF1 mutants were generated by site-directed mutagenesis using pET28-MHL-NRF1 (aa 54–284) and (aa 177–284) expression plasmids as templates. All the recombinant plasmids were expressed in *Escherichia coli* BL21 (DE3) and induced with 0.5 mM IPTG overnight at 14 °C. The cells were collected and resuspended in a lysis buffer containing 500 mM NaCl, 20 mM Hepes (pH 7.0) and 5% glycerol. The cell lysates were then sonicated at 4 °C. After centrifugation at 16000 g, the supernatants were applied to Ni-NTA resin (Qiagen) for purification. The recombinant protein was eluted and subsequently treated with TEV protease to remove the His_6_-tag. The wild-type NRF1 and mutants were purified by affinity chromatography in a buffer containing 150 mM NaCl and 20 mM Hepes (pH 7.0). The purified protein was diluted using buffer A (20 mM Hepes, pH 7.0) and subjected to anion-exchange chromatography analysis with a gradient of 0–1000 mM NaCl. For gel-filtration column chromatography, the buffer consisted of 20 mM Hepes (pH 7.0) and 150 mM NaCl. The purified wild-type and mutant NRF1 proteins were concentrated to a concentration of 10–20 mg/mL using concentrators (Millipore, Amicon® Ultra-15 Centrifugal Filter) and stored in a storage buffer consisting of 20 mM Hepes (pH 7.0) and 150 mM NaCl.

### Isothermal titration calorimetry (ITC) assay

Isothermal titration calorimetry (ITC) assays were conducted to assess the binding ability of NRF1 to double-strand DNA (dsDNA) at 25°C using the MicroCal PEAQ-ITC instrument (Malven). The DNA oligonucleotides were procured from General Biosystems Co. Ltd. (Anhui) and subsequently annealed to form DNA duplexes in a buffer consisting of 20 mM Hepes (pH 7.0) and 150 mM NaCl. For the ITC binding assays, the final concentrations of proteins and dsDNAs were ranging from 20 to 30 μM and 500 to 600 μM, respectively. The dissociation constant (*K*_d_) was calculated using the ‘One Set of Sites’ fitting model, employing a nonlinear least-squares method in the MicroCal ITC200 analysis software Origin 7.0 (Malven). The ITC assays were measured in triplicate, and the *K*_d_ values were the averages of the three measurements. For the samples with nearly undetectable binding ability, most of the ITC experiments were conducted twice.

### Size exclusion chromatography-multiangle light scattering (SEC-MALS) analysis

To analyze the oligomeric state of NRF1, we designed several constructs, including aa 54–100, aa 54–177, aa 98–177, aa 98–149, aa 128–177, aa 177–284 and aa 54–284. All of them were purified as the methods above. Among these constructs, only aa 54–177, aa 98–177, aa 177–284 and aa 54–284 yielded soluble and stable proteins. Subsequently, we performed SEC-MALS analyses for all these soluble proteins. The SEC-MALS experiments were performed using an HPLC-MALS system. A DAWN TREOS multiangle light scattering detector and an Optilab T-rEX refractometer were used in line with the WTC-015S5 hydrophilic film bonded silica column (Wyatt Technology). The column was pre-equilibrated with a buffer consisting of 50 mM PBS and 150 mM NaCl at a flow rate of 0.5 mL/min. Molecular weights of proteins were determined using a dn/dc value (refractive index increment) of 0.185 mL/g and the Astra 6.1 program developed by Wyatt Technology.

### Differential scanning fluorometry (DSF) analysis

In this study, differential scanning fluorometry (DSF) analyses were carried out to evaluate the protein stability using a real-time PCR instrument (Light Cycler 480, Roche, Switzerland). The protein samples (1.5 mg/mL) were prepared in a buffer containing 20 mM Hepes (pH 7.0) and 150 mM NaCl, with the fluorescent dye SYPRO Orange (Sigma) added at a final concentration of 16×. A total volume of 10 μL of the mixture was used for measuring the melting curves. The temperature was raised gradually from 25°C to 90°C at a rate of 2°C/min. The excitation wavelength and emission wavelength used for the fluorescence measurements were 465 and 580 nm, respectively. All experiments were performed in triplicate. The melting temperatures (Tms) were calculated using LightCycler Thermal Shift Analysis (Light Cycler 480, Roche), and melting curves were prepared using GraphPad Prism 9.0.

### Protein crystallization

To obtain the crystals of the NRF1-dsDNA complex, the NRF1 protein samples (10 mg/mL) were mixed with dsDNAs at a molar ratio of 1:1.2. The protein-dsDNA complex was mixed with the Hampton Research Screen kits, and crystallization was then set up using the sitting-drop vapor diffusion method at 18°C. Crystals of the NRF1 dimer (aa 54–284) bound to 14bp ATGCGCATGCGCAT dsDNA were grown using a reservoir solution consisting of 2% (v/v) Tacsimate™ (pH 6.0), 0.1 M Bis-Tris (pH 6.5), and 20% (w/v) Polyethylene glycol 3350 after three days. Crystals of the NRF1 DBD (aa 177–284) in complex with 16bp GGTGCGCATGCGCACC dsDNA were grown in a reservoir solution consisting of 0.2 M Ammonium sulfate, 0.1 M Bis–Tris (pH 6.5) and 25% (w/v) Polyethylene glycol 3350 after two days. Before data collection, the crystals were safeguarded with a cryoprotectant composed of the crystallization condition supplemented with an additional 20–25% (v/v) glycerol or ethylene glycol. Subsequently, the crystals were flash-frozen in liquid nitrogen.

### Data collection and structure determination

Diffraction data for the NRF1 dimer-14 bp dsDNA complex were collected at SSRF 02U1 beamline at 100 K, and processed with the XDS (22) and CCP4 (23). For the NRF1 DBD-16bp dsDNA complex, the diffraction data were collected using an in-house Rigaku XtaLAB Synergy Custom instrument at 100 K. The data were processed using Rigaku CrysAlisPro. The structure of the NRF1 dimer-dsDNA complex was solved by the molecular replacement with the program PHASER (24) using the AlphaFold2 predicted NRF1 structure model as the search model (25,26). During the process of molecular replacement, we were able to successfully find the position of one NRF1 monomer (monomer1) and the DNA binding domain of another monomer (monomer2). Further manual model building based on electron density allowed us to build the remaining portion (the dimerization domain) of monomer2. To solve the structure of the NRF1 DBD–dsDNA complex, the structure model of NRF1 from the NRF1 dimer–dsDNA complex structure was used as the search model. Model building was carried out using Coot (27), and structure refinement was performed using PHENIX.REFINE (28). The diffraction data collection and refinement statistics are summarized in Supplementary Table S1.

### Cell culture and western blot

The NRF1 wild-type and mutants, including the DD truncation (deleted aa 96–176), DBD truncation (deleted aa 177–284), and DNA base-interacting residue mutants, were subcloned into pcDNA3.1, respectively. The HEK293T cells were cultured in Dulbecco's modified Eagle's medium (DMEM) high-glucose media (Cytiva) supplemented with an additional 0.1 × Penicillin-Streptomycin and 10% fetal bovine serum (Hyclone) in 5% CO_2_ at 37°C. Twenty-four hours after transfection, the cells expressing Flag- and His_10_-tagged NRF1 wild-type or mutants were washed with ice-cold PBS. The cells were then lysed and subjected to SDS-PAGE analysis. The proteins were transferred to a PVDF membrane, which was subsequently blocked with 5% milk in PBST for one hour at room temperature. The membrane was then incubated with anti-Flag (Cat no: 66008-4-Ig, proteintech) and anti-GAPDH (Cat no: 60004–1-Ig, proteintech) primary antibodies overnight at 4°C. The membrane was washed with TBST three times, and a secondary antibody conjugated with HRP (Cat no: SA00001-1, proteintech) was applied. Following another round of washing with TBST, the protein samples were detected using an ECL Western blotting detection reagent (Meilunbio) and exposed to a Multicolor fluorescent gel imaging system (FluorChem R).

### Luciferase reporter assay

The pGL4.20 vector encodes a luciferase reporter gene luc2 (Photinus pyralis) and is commonly employed in a luciferase assay to measure the transcription activity regulated by transcription factors. The different promoter regions of *TFAM* were individually subcloned into a pGL4.20 vector (MiaoLing Biology). Additionally, the *TFAM* promoter regions spanning −201 to + 104 transcription start site (TSS) with different GCGCCTGCGC mutation motifs were designed based on our ITC binding data, followed by individually subcloning into the pGL4.20 vector. Subsequently, HEK293T cells were co-transfected with pcDNA3.1-NRF1 WT or pcDNA3.1-NRF1 mutant plasmids (400 ng, pcDNA3.1 plasmid served as the control), pGL4.20-*TFAM*-promoter WT or its mutant plasmids (75 ng, pGL4.20 plasmid served as the control), and pRL-TK (5 ng, Renilla-luciferase expressing plasmid used as an internal control) using Lipofectamine 2000 (Invitrogen). Twenty-four hours after transfection, the cells were washed with ice-cold PBS and then lysed using passive lysis buffer (Promega). Luciferase activity was measured using the Dual-Luciferase reporter assay system (Promega). Normalized luciferase activity was determined using the ratio of firefly luciferase activity to Renilla luciferase for each sample. The measurements were performed using a GloMax 20/20 Luminometer (Promega) in triplicate (*n* = 3), and the Luciferase activity values were the averages of the measurements, which were analyzed using one-way ANOVA by GraphPad Prism 9.0.

## Results

### NRF1 homodimer specifically binds to the consensus DNA sequence GCGCATGCGC

Previous studies have demonstrated that NRF1 functions as a homodimer and binds to the consensus DNA sequence GCGCNTGCGC (N represents any nucleotide) identified in the promoter regions of different NRF1 target genes (Figure 1A and B) (4,^10^,^29^,^30^). To explore the oligomeric state and the binding ability of NRF1, we subcloned a series of NRF1 fragments based on the AlphaFold2 predicted NRF1 model and the reported DNA binding data of NRF1 (5,25) (Supplementary Figure S1A). It has been previously reported that the N-terminus of NRF1 (aa 1–304) is involved in DNA recognition (5). We first analyzed the oligomeric state of NRF1 using size exclusion chromatography-multiangle light scattering (SEC-MALS). Our SEC-MALS results showed that the NRF1 fragments, including NRF1 (aa 54–284), NRF1 (aa 54–177) and NRF1 (aa 98–177), exist as a dimer with an average mass of ∼52.8, 28.1 and 18.5 kDa in solution, respectively, while the NRF1 (aa 177–284) fragment displays an average molecular weight of ∼12.3 kDa corresponding to a monomer, suggesting that the NRF1 (aa 54–177) fragment, especially the NRF1 (aa 98–177) region, mediates the dimerization of NRF1 (Supplementary Figure S1B). Considering the degradation of the full-length NRF1, we performed the isothermal titration calorimetry (ITC) assays using the NRF1 construct of aa 54–284 that yields a soluble and stable protein. Our ITC results displayed that NRF1 (aa 54–284) binds to an ATGCGCATGCGCAT dsDNA with a *K*_d_ of ∼0.3 μM, which is ∼3-fold stronger than the ATGCGCCGGCGCAT dsDNA, meaning that NRF1 demonstrates a clear preference for the central dinucleotide sequence AT over CG within the GCGCNTGCGC motif (Figure 1C, Table 1, Supplementary Figure S2). To explore whether the 14 bp dsDNA is long enough for the NRF1 binding, we performed an additional ITC binding assay using a longer 20 bp GCGATGCGCATGCGCATCGC dsDNA. Our ITC data demonstrated that the NRF1 (aa 54–284) binds to the 20bp DNA with similar binding affinity as that observed for the 14bp AT central dsDNA (Supplementary Figure S2), suggesting that the 14bp dsDNA is long enough for NRF1 binding.

**Table 1.** Binding affinities of NRF1 (aa 54–284) to dsDNAs containing different numbers of nucleotides as the central spacer between the two GCGC half-sites

DNA sequences	*K*_d_ (μM)
5′-ATGCGC**AT**GCGCAT-3′(WT)	0.31 ± 0.05
5′-ATGCGC**CG**GCGCAT-3′	1.09 ± 0.06
5′-ATGCGCGCGCAT-3′	0.57 ± 0.02
5′-ATGCGC**A**GCGCAT-3′	1.62 ± 0.11
5′-ATGCGC**ACT**GCGCAT-3′	1.96 ± 0.27
5′-ATGCGC**ACGT**GCGCAT-3′	0.91 ± 0.04
5′-ATGCGC**ACACT**GCGCAT-3′	2.32 ± 0.48
5′-ATGCGC**ACACTT**GCGCAT-3′	4.36 ± 0.29
5′-ATGCGC**ACACTCT**GCGCAT-3′	4.31 ± 0.28

Only one strand of the DNA duplex is shown in the table, and the nucleotides of the central spacer are bold.

Although NRF1 has been reported to recognize the GCGCNTGCGC sequence with the 2 bp central spacer between two palindromic GCGC half-sites (6), we wondered whether the number of spacing nucleotides between two GCGC half-sites affects the binding ability of NRF1. Thus, we conducted ITC assays to measure the binding ability of NRF1 (aa 54–284) with dsDNAs that contain different numbers of central spacer between two GCGC half-sites. Our ITC data showed that dsDNA with 1bp or no central spacer between the GCGC half-sites exhibited ∼2–5 fold reduced binding affinity to NRF1 compared to the AT central dsDNA. Similarly, when the number of spacing nucleotides was increased to 3–7 bp, the dsDNAs also exhibited ∼3–14-fold decreased binding affinity to NRF1 (Table 1, Supplementary Figure S2). Furthermore, to investigate the sequence selectivity of the GCGC motif, we designed a series of DNA oligonucleotide mutants derived from the ATGCGCATGCGCAT sequence and evaluated their binding ability to the NRF1 (aa 54–284). We found that mutating any nucleotide in the GCGC repeats led to varying degrees of reduction in binding affinities to NRF1 (Table 2, Supplementary Figure S3). Taken together, these results suggest that NRF1 is capable of binding to dsDNA with different numbers of central spacer between two GCGC half-sites, but it exhibits a binding preference for the consensus sequence GCGCATGCGC.

**Table 2.** Binding affinities of NRF1 (aa 54–284) to different dsDNA variants

DNA sequences	*K*_d_ (μM)
5′-ATGCGCATGCGCAT-3′*(WT)	0.31 ± 0.05
5′-ATaCGCATGCGCAT-3′	1.27 ± 0.27
5′-ATaCGCATGCGtAT-3′	WB
5′-ATtCGCATGCGCAT-3′	2.11 ± 0.80
5′-ATtCGCATGCGaAT-3′	WB
5′-ATGaGCATGCGCAT-3′	1.38 ± 0.15
5′-ATGaGCATGCtCAT-3′	WB
5′-ATGtGCATGCGCAT-3′	1.81 ± 0.25
5′-ATGtGCATGCaCAT-3′	WB
5′-ATGCaCATGCGCAT-3′	0.66 ± 0.04
5′-ATGCaCATGtGCAT-3′	4.43 ± 0.55
5′-ATGCtCATGCGCAT-3′	2.23 ± 0.17
5′-ATGCtCATGaGCAT-3′	WB
5′-ATGCGaATGCGCAT-3′	3.75 ± 0.69
5′-ATGCGaATtCGCAT-3′	WB
5′-ATGCGtATGCGCAT-3′	1.25 ± 0.11
5′-ATGCGtATaCGCAT-3′	1.31 ± 0.18
5′-ATattaATGCGCAT-3′	3.76 ± 0.23

*: The DNA sequence used for crystallization in this study. Only one strand of the DNA duplex is shown in the table, and the mutated nucleotides are shown in lower case. WB: weak binding.

### Crystal structure of the NRF1 homodimer in complex with the GCGCATGCGC dsDNA

**Figure 2.**
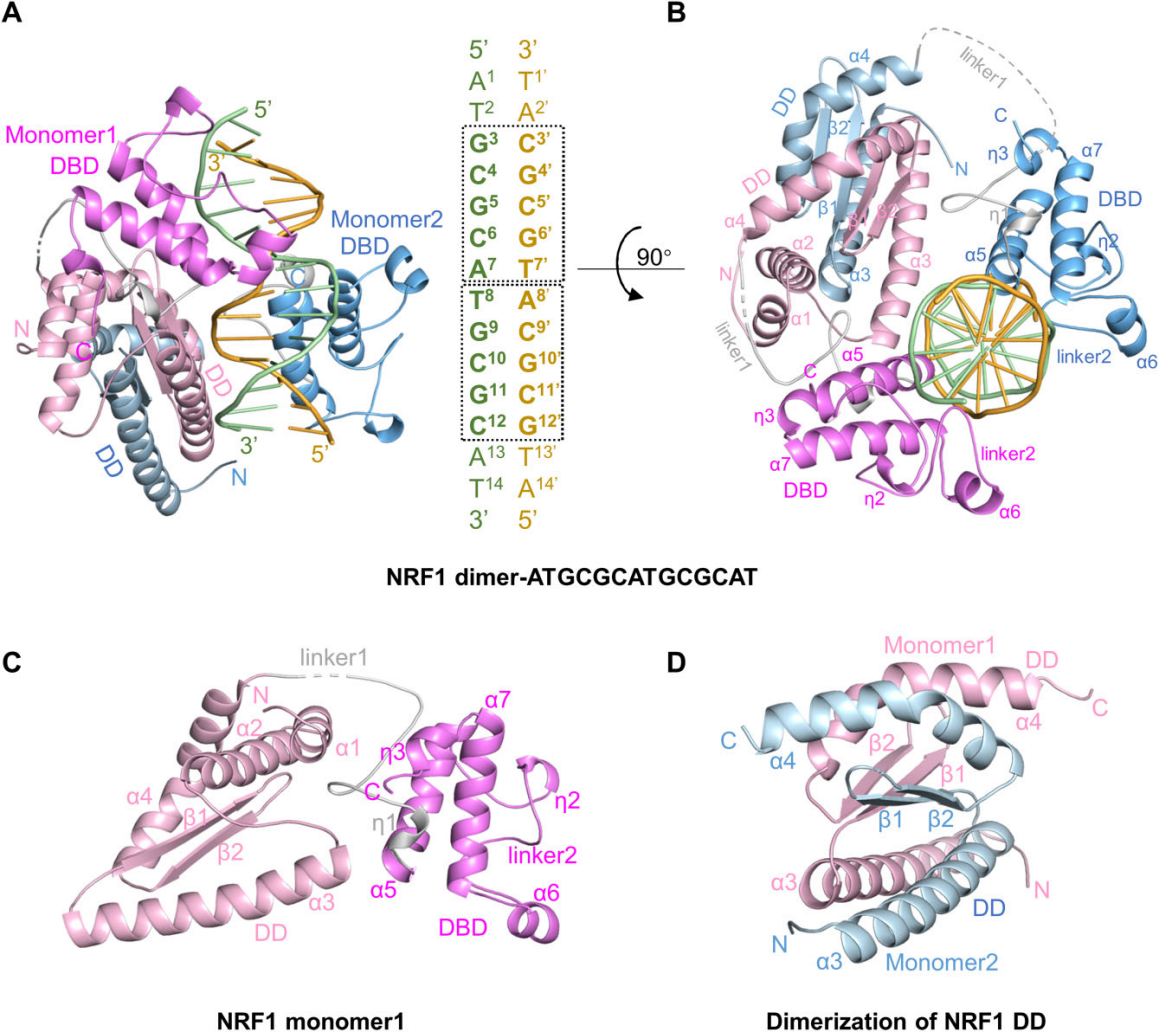
Crystal structure of the NRF1 homodimer bound to dsDNA with a sequence of ATGCGCATGCGCAT. **(A)** Overall structure of the NRF1 homodimer in complex with the ATGCGCATGCGCAT dsDNA. The two NRF1 monomers are colored in pink and blue, respectively, with the DNA binding domain highlighted in dark pink and deep blue, respectively. The linker1 is colored gray. DNA is shown in cartoon mode with one strand colored yellow and the other one green. The boxes indicate the positions of two TGCGC sequence repeats. From the DNA 5′ to 3′, one repeat is the TGCGC sequence, while the other is the GCGCA sequence that is complementary to the TGCGC sequence of the other DNA strand. In the figures and the text, one DNA strand is numbered following the 5′ to 3′ numbering of the oligo, while the complementary DNA strand has the same numbering as its pairing bases with a prime sign to distinguish the two strands. **(B)** Structure of the NRF1 homodimer in complex with dsDNA viewed from the top of Figure 2A. **(C)** Structure of dimerization domain (DD, pink) and DNA binding domain (DBD, dark pink) of the NRF1 monomer1. **(D)** The dimeric structure formed by NRF1 DDs. DBDs are omitted for clarity.

To investigate the molecular mechanisms underlying the selective binding of NRF1 to the GCGCATGCGC DNA sequence, we determined the crystal structure of NRF1 (aa 54–284) with a 14pb ATGCGCATGCGCAT dsDNA (Supplementary Table S1). In the complex structure, NRF1 forms a homodimer and binds to a single ATGCGCATGCGCAT DNA duplex (Figure 2A and B, Supplementary Figure S4A). Each NRF1 monomer comprises a dimerization domain (DD, residues 54 to 172) followed by a DNA binding domain (DBD, residues 201–284), and the two domains are connected by a flexible linker (aa 173–201, named linker1, the loop consisting of aa 218–249 in the DBD are named linker2 hereafter) (Figure 2C). Strikingly, the electron density of the N-terminal 43 residues (aa 54–94) in the NRF1 monomer2 and part of the linker1 from both monomers are not visible, suggesting the structural flexibility of these regions. Structural analysis shows that the space where the first two helices α1 and α2 of monomer2 are expected to be located is occupied by a symmetry-related molecule, suggesting that the first two helices of monomer2 might assume different conformation or be proteolyzed in the crystal. In addition, the flexible linker1 between the two domains might explain why NRF1 could bind to dsDNA with different numbers of central spacer between the two GCGC half-sites (Table 1, Supplementary Figure S2).

The DD consists of four helices α1 to α4, along with two antiparallel β-strands β1 and β2. The β1 and β2 strands from each monomer form an intermolecular 4-stranded β-sheet, while the α3 and α4 helices from two monomers form antiparallel helices and pack against either side of the β-sheet through extensive hydrophobic interactions (Figure 2D, Supplementary Figure S4B and S4C). In this way, two NRF1 form a tightly intertwined homodimer via the DD, consistent with our SEC-MALS results that the fragment of residues 98–177 is necessary for the dimerization of NRF1 (Supplementary Figure S1B). The DBD is composed of three helices α5 to α7 and two short 3_10_-helix, and each DBD possesses positively charged surfaces to recognize two neighboring major grooves of the DNA duplex from nearly opposite side (Figure 2A–C, Supplementary Figure S4A and S4C).

Interestingly, in the NRF1 dimer-dsDNA complex, although the DD or DBD from the two monomers superimposes very well (with RMSDs of 0.33Å and 0.34Å, respectively), the arrangement of these two domains differs in both monomers (Supplementary Figure S4D–S4F). In monomer1, the DD and DBD are situated on the same side of the DNA duplex. However, in monomer2, the DBD and DD adopt a U-turn conformation, with its DD dimerizing with the DD from monomer1 on one side, and the DBD binding to the major groove of the DNA duplex from nearly the opposite side (Figure 2A and B, Supplementary Figure S4A). To explore whether the DNA binding mode of NRF1 is novel, we queried the DALI and FACTCA servers. Both queries revealed that serum response factor (SRF) is the closest structural homolog of NRF1, with a *Z*-score of 5.9 and a *P*-value of 8.66e-5, respectively (31,32). SRF is a transcription factor that belongs to the MADS domain protein family, playing key roles in cell development and cell migration (33–35). Structural comparison of SRF and NRF1 shows that the SRF MADS domain and the NRF1 DD form a dimeric interface in a similar manner (Supplementary Figure S5A and S5B). However, SRF does not have a specific DNA binding domain, it binds to the serum response element (SRE) of the promoters via its MADS domain (36–38), indicating that the DNA binding mode exhibited by the NRF1 homodimer is novel.

### Structural basis for specific recognition of the GCGCATGCGC sequence by NRF1

**Figure 3.**
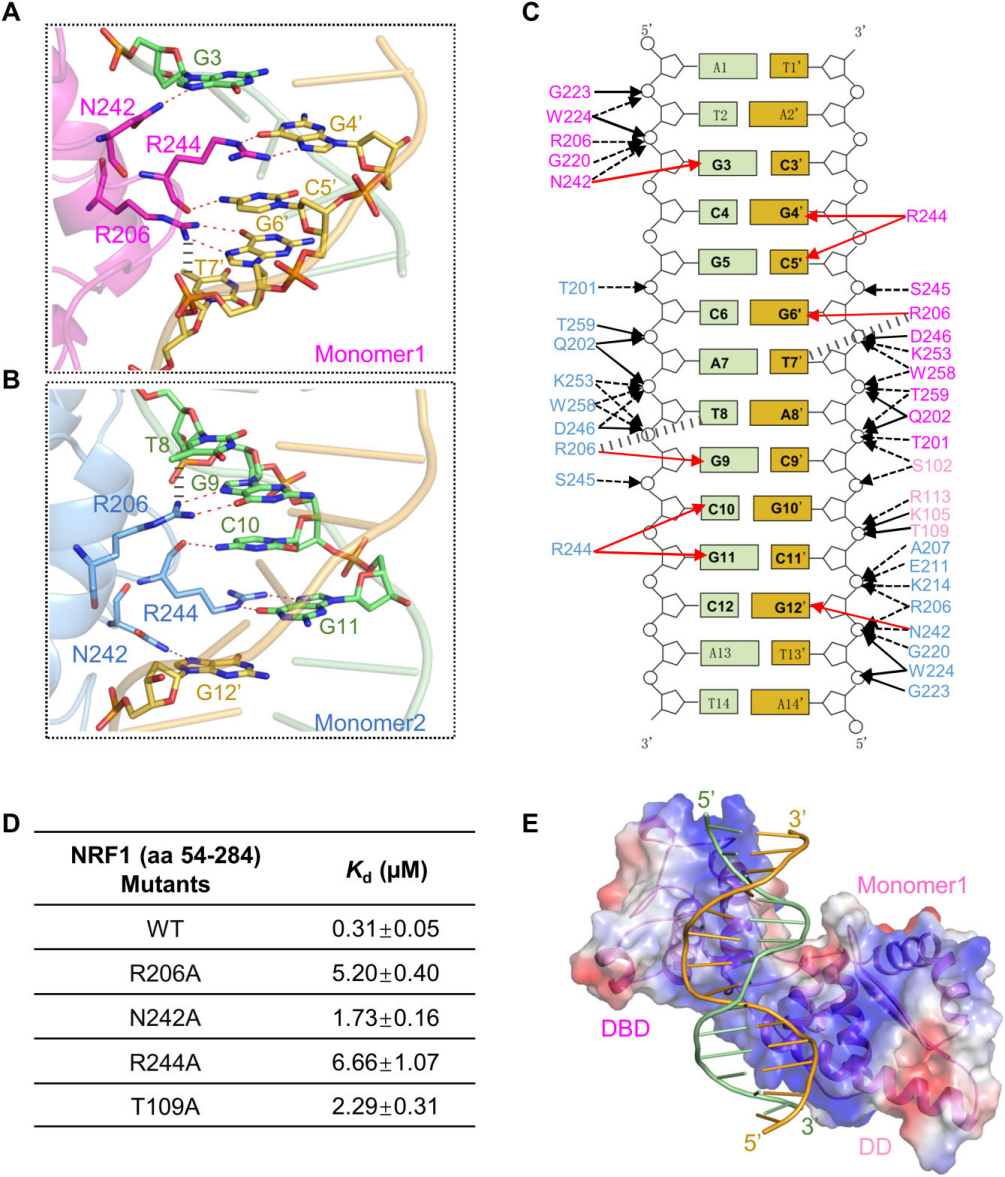
Structural basis for preferential recognition of the GCGCATGCGC dsDNA by the NRF1 homodimer. **(A** and **B)** Detailed base interactions between the NRF1 DBD and the ATGCGCATGCGCAT dsDNA. The protein residues and dsDNA bases are shown in stick models. Hydrogen bonds formed between protein and dsDNA are marked as red dashed lines, while the cation-π interactions between residue R206 and base T8 or T7′ are shown in gray dashed lines. **(C)** Schematic diagram of the NRF1 homodimer binding to the ATGCGCATGCGCAT dsDNA. The two conserved TGCGC motifs are highlighted in black fonts. The direct base and phosphate group interactions are marked as red and black solid arrows, respectively, while the cation-π interaction and water-mediated hydrogen bonds are indicated by gray dashed lines and black dashed arrows, respectively. The numbering of dsDNA is in the same way as Figure 2A. **(D)** Binding affinities of the NRF1 mutants to the ATGCGCATGCGCAT dsDNA. **(E)** NRF1 DD forms interactions with the DNA duplex. The NRF1 monomer1 is represented as a transparent electrostatic surface and dsDNA is shown in a cartoon mode.

In the NRF1 dimer-dsDNA complex, two DBDs of the NRF1 dimer bind to the DNA duplex from opposite directions. The helix α5 and a long linker2 (aa 218–249) connecting α5 and α6 from one DBD insert into the major groove of the DNA duplex from one side, specifically recognizing the first TGCGC motif (i.e. G3C4G5C6A7). The helix α5 and the linker2 of the other DBD insert into the adjacent major groove of the DNA duplex from the other side, allowing it to recognize the second TGCGC motif (i.e. T8G9C10G11C12) (Figures 2A, B, 3A and B). Specifically, in NRF1 monomer1, the side chain of N242 in linker2 forms a hydrogen bond with the G3, while the side chain and main chain carbonyl group of R244 from linker2 form three hydrogen bonding interactions with the bases of G4′ and C5′ that are complementary with C4 and G5, respectively. The side chain of R206 forms bidentate hydrogen-bonding interactions with the base of G6′, and simultaneously forms cation-π interaction with the pyrimidine ring of T7′ (Figure 3A and C). As a result, the DBD of the NRF1 monomer1 contributes to the sequence specific-binding to the C3′G4′C5′G6′T7′ sequence that pairs with the first TGCGC motif (Figure 3A and C). This binding mode is conserved in monomer2. The side chain of R206 forms two hydrogen bonding interactions with G9 and also engages in cation–π interaction with the pyrimidine ring of T8. The N242 and R244 from linker2 form specific hydrogen bonding interactions with the C10, G11, and G12′ that pairs with C12. Thus, the three residues R206, N242 and R244 of monomer2 contribute to the specific binding of the second TGCGC motif in a similar manner (Figure 3B and C). To investigate the significance of these three residues in DNA binding, we mutated R206, N242 or R244 to alanine individually, and examined their DNA binding ability by ITC assays. Our ITC results showed that mutating R206, N242 or R244 to alanine reduces the binding affinity by ∼5- to 22-fold compared to the wild-type NRF1 (Figure 3D, Supplementary Figure S6). Hence, residues R206, N242, and R244 of NRF1 DBD are critical in sequence-specific binding to the GCGCATGCGC motif.

Apart from base-specific recognition, residues T201, Q202, R206, G220, G223, W224, N242, S245, D246, K253, W258 and T259 from each NRF1 monomer form extensive direct and water-mediated interactions with the backbones of the DNA duplex in almost identical manners (Figure 3C). Remarkably, the helix α3 from the DD of the NRF1 monomer1 exhibits a positively charged surface and forms electrostatic interaction with the DNA duplex (Figure 3E). This interaction is further stabilized by the hydrogen bonds formed between the residues S102, K105, T109 and R113 from helix α3 and the phosphate groups of the A8′, C9′ and G10′, respectively (Figure 3C, Supplementary Figure S5C). To assess the binding ability of the NRF1 DD, we then carried out an ITC assay using the 14 bp ATGCGCATGCGCAT dsDNA and NRF1 (aa 54–177). ITC data showed that the NRF1 DD alone is capable of binding 14bp DNAs with a *K*_d_ of ∼1.8 μM, albeit ∼6-fold weaker than the NRF1 dimer (Supplementary Figure S5D). In addition, mutating residue T109 of the NRF1 (aa 54–284) to alanine decreased the binding affinity by ∼7-fold compared to the wild-type NRF1 (Figure 3D, Supplementary Figure S7). Taken together, in the NRF1 dimer-dsDNA complex, the NRF1 DBDs bind to the major grooves of the DNA duplex and contribute to sequence-specific binding, while one of the DDs forms the interactions with the DNA duplex, the cooperatively of DD and DBD stabilizes the NRF1–dsDNA complex.

### The consensus binding sequence GCGCATGCGC of NRF1 is verified by mutagenesis

To investigate the importance of the GCGCATGCGC consensus sequence in NRF1 binding, we generated a series of dsDNA variants based on the ATGCGCATGCGCAT sequence and performed ITC binding assays with the NRF1 dimer. Our ITC data demonstrated that substituting G3 with either A or T reduces its binding affinity by ∼4- or ∼7-fold, respectively (Table 2, Supplementary Figure S3). Simultaneous mutation of both G3 and its palindromic nucleotide C12 further decreases the binding affinity (Table 2, Supplementary Figure S3). Our structure models explain why the nucleotide mutations reduce their binding to NRF1. When substituting G3 with A or T, the hydrogen bond between the side chain of N242 and the N7 atom of G3′ is disrupted, and a steric clash is introduced between the side chain of N242 and the methyl group of the T (Supplementary Figure S8A and S8B). Although we did not carry out ITC assay for the G3 to C mutation, our structural model indicates that a similar structural change would be observed when G3 is replaced with C, suggesting that the G3 to C mutation would also reduce its binding affinity to NRF1 (Supplementary Figure S8C).

Substituting C4 with A or T results in a binding affinity reduction by ∼4 or ∼6-fold, respectively (Table 2, Supplementary Figure S3). Similarly, simultaneous mutation of both C4 and its palindromic nucleotide G11 leads to a further decrease in binding affinity (Table 2, Supplementary Figure S3). Structural analysis reveals that replacing C4 with A, T or G disrupts the bidentate hydrogen-bonding interactions between the side chain guanidine group of R244 and the O6 and N7 atoms of G4′, and a steric clash is also introduced between the side chain of R244 and T4′ or C4′, respectively (Supplementary Figure S8D-S8F).

We also examined the role of G5 in the 14bp dsDNA. Mutating G5 to either A or T reduces the binding affinity by ∼2- or ∼7-fold, respectively, compared to the wild-type DNA (Table 2, Supplementary Figure S3). When both G5 and its palindromic nucleotide C10 were simultaneously mutated to A and T to create the ATGCaCATGtGCAT dsDNA, NRF1 still exhibits a binding affinity of ∼4 μM. However, when they are simultaneously mutated to T and A (ATGCtCATGaGCAT), the binding affinity is significantly diminished (Table 2, Supplementary Figure S3). Our structural modeling shows that when G5 is mutated to A, T or C, the hydrogen bond between the carbonyl group of R244 and the N4 atom of C5′ that pairs with G5 is disrupted. However, the side chain of R244 forms a cation-π interaction with the pyrimidine ring of T5′ that pairs with A5 (Supplementary Figure S8G) (39,40). Nevertheless, this stacking interaction is not observed when G5 is mutated to T or C (Supplementary Figure S8H and S8I), explaining why mutating G5 to A still maintains a binding affinity to NRF1.

The DNA variants with C6 mutated also exhibit reduced binding affinities (Table 2, Supplementary Figure S3). Our ITC data showed that replacing C6 with A or T reduces the binding affinity by ∼12- or ∼4-fold, respectively. Additionally, when C6 and its palindromic G9 are simultaneously mutated to A and T to create the ATGCGaATtCGCAT sequence, this DNA variant almost loses its binding ability to NRF1. In contrast, simultaneously mutating C6 and its palindromic G9 to T and A (ATGCGtATaCGCAT), it still exhibits a binding affinity of ∼1.3 μM (Table 2, Supplementary Figure S3). Structural analysis reveals that when replacing C6 with A, T6′ that pairs with A6 not only loses the bidentate hydrogen-bonding interactions between the side chain guanidine group of R206 and the O6 and N7 atoms of G6′ but also clashes with the side chain of R206 (Supplementary Figure S8J). Conversely, when C6 is mutated to T, the side chain of R206 still forms a hydrogen bond with the N7 atom of A6′ that pairs with T6 (Supplementary Figure S8K). Similarly, the hydrogen bonds between the R206 and G6′ are disrupted when C6 is substituted with G6, suggesting that mutation of C6 to G would also reduce the binding affinity of NRF1 (Supplementary Figure S8L).

Our structure also explains the preference of the NRF1 dimer for the central AT dinucleotide (Figure 1C and Supplementary Figure S3). In addition to the hydrogen bonding interactions with the guanine bases, the side chains of R206 from both NRF1 monomers also form cation-π interactions with the pyrimidine ring of T8 and T7′ that pairs with A7, respectively (Figure 3A–C). When the central AT is replaced with a CG dinucleotide, the cation-π interactions are disrupted. The TpG dinucleotide recognition pattern in the TGCGC half-site sequence, in which the side chain of the R206 simultaneously forms bidentate hydrogen-bonding interactions with the guanine base and stacks with the thymine base, is also observed in other DNA binding proteins, which typically play roles in the sequence-specific recognition of protein (41). Collectively, our mutagenesis and binding assays, combined with structural analysis, underscore the crucial roles of the GCGCATGCGC DNA sequence in NRF1 binding.

### Cooperation of DD and DBD is required for binding of the intact GCGCATGCGC DNA sequence by NRF1

**Figure 4.**
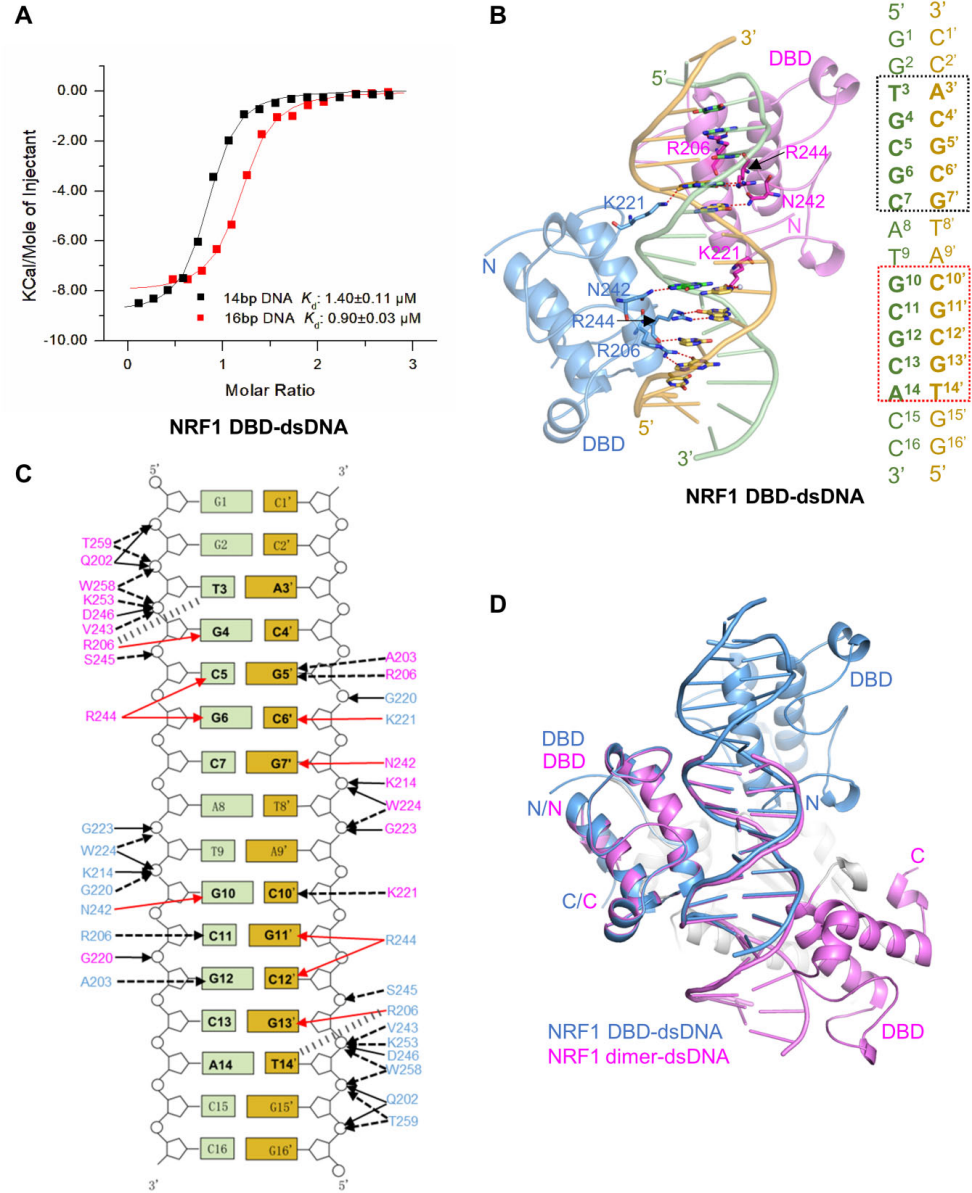
Cooperation of DD and DBD is required for binding of the intact GCGCATGCGC DNA sequence by NRF1. **(A)** ITC binding curves of NRF1 DBD (aa 177–284) to 14bp ATGCGCATGCGCAT and 16bp GGTGCGCATGCGCACC dsDNA, respectively. Only one strand of the DNA duplex is displayed. **(B)** Structure of the NRF1 DBD in complex with 16pb GGTGCGCATGCGCACC dsDNA. Both NRF1 DBDs are shown in a cartoon representation colored pink and blue, respectively, and the DNA duplex is shown in the same way as in Figure 2A. The protein residues and DNA bases are shown in stick models. Hydrogen bonds formed between protein and DNA base are marked as red dashed lines. **(C)** Schematic diagram of both NRF1 DBDs binding to the GGTGCGCATGCGCACC dsDNA. The two conserved TGCGC motifs are highlighted in black fonts. The interactions between the DBD and dsDNA are shown in the same way as in Figure 3C. The numbering of dsDNA is in the same way as Figure 2A. **(D)** Structural alignment of the NRF1 DBD-16bp dsDNA complex (blue) with the NRF1 homodimer–dsDNA complex (pink).

Considering that the linker1 connecting the DD and DBD is partially disordered in our NRF1 dimer-dsDNA structure, and that the DBD alone exists as a monomer in the SEC-MALS analysis, we asked if the DBD itself could bind DNA tightly. To investigate this, we expressed the NRF1 DBD fragment (aa 177–284) alone and assessed its binding affinity to the 14bp dsDNA that we used for crystallization. Our ITC data showed that the NRF1 DBD binds to this dsDNA with a *K*_d_ of ∼1.4 μM, albeit ∼5-fold weaker than the NRF1 dimer (Figure 4A, Supplementary Figure S9A). This finding indicates that the DBD alone has a slightly diminished binding affinity for the ATGCGCATGCGCAT dsDNA, which is consistent with our observation that the cooperation of DD and DBD is required for the DNA binding of NRF1.

Subsequently, we successfully determined one complex structure of the NRF1 DBD bound to a 16bp dsDNA with a sequence GGTGCGCATGCGCACC (Supplementary Table S1). This 16bp dsDNA exhibits a comparable binding affinity to the NRF1 DBD as the 14bp dsDNA (Figure 4A, Supplementary Figure S9A). In the NRF1 DBD–16pb dsDNA complex, only one NRF1 DBD molecule and a single DNA strand are observed in an asymmetric unit (ASU), and the complementary DNA strand from another ASU binds to another NRF1 molecule. In addition to the interactions between the NRF1 molecule and the DNA in one ASU, its symmetry-related NRF1 molecule from the other ASU also contributes to the electrostatic and base interactions with the DNA duplex, which results in two NRF1 DBDs bound to a DNA duplex. Structural analysis shows that the residues R206, N242 and R244 from each DBD molecule interact specifically with one TGCGC motif, similar to what is observed in the NRF1 dimer-dsDNA complex structure (Figure 4B and C). Interestingly, residues K221 from the linker2 insert into the minor grooves to form direct or water-mediated interactions with C6′ and C10′, respectively (Figure 4B and C). Mutations of any of these residues of DBD led to a significant reduction or complete loss of binding between NRF1 DBD and the 16bp dsDNA (Supplementary Figure S9).

Structural comparison of the NRF1 DBD-dsDNA and NRF1 dimer-dsDNA complexes shows that although the DNA base interactions are almost the same, the two DBDs exhibit distinct arrangements in the two complexes (Figure 4D). In the NRF1 DBD-dsDNA complex, the two DBD molecules bind separately to the two TGCGC motifs of the GGTGCGCATGCGCACC DNA, approaching the DNA duplex from nearly the same side with a tail-to-tail-like orientation (Figure 4B-D). However, in the NRF1 dimer-dsDNA complex, the DD restricts the orientation of the DBD, enabling the two DBDs to bind to the intact GCGCATGCGC motif from nearly the opposite sides of the DNA duplex in a head-to-head orientation (Figure 2B and 4D). Therefore, these findings illustrate that although the NRF1 DBD alone is capable of binding to the TGCGC motif, the cooperation of DD and DBD is required for the intact GCGCATGCGC sequence binding of NRF1.

### NRF1 dimerization and an intact consensus sequence GCGCNTGCGC are indispensable for the transcriptional activity of NRF1

**Figure 5.**
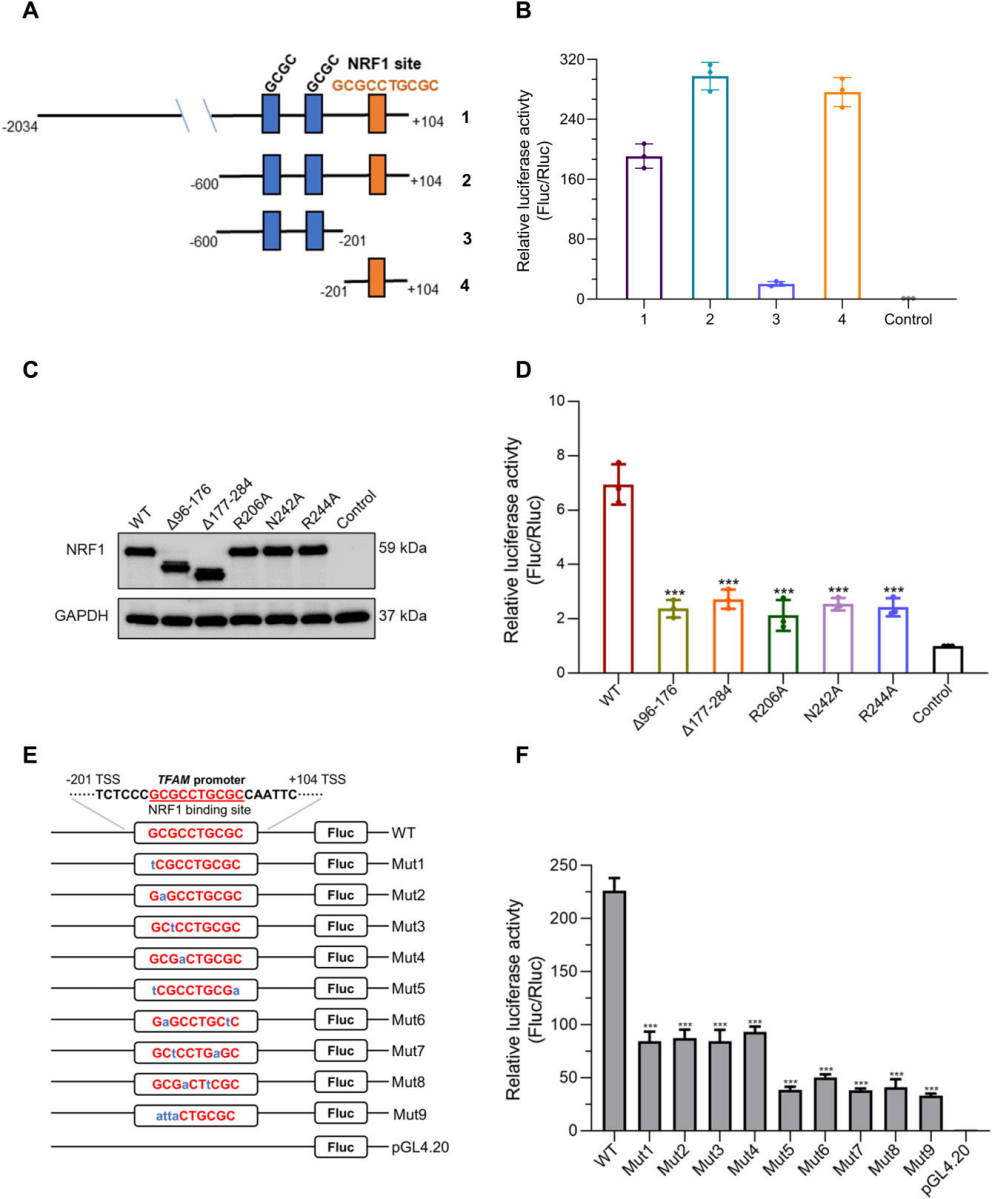
NRF1 dimerization and an intact GCGCCTGCGC sequence are indispensable for the *TFAM* activation by NRF1. **(A)** Schematic representation of the designed human *TFAM* promoter constructs. The schematic includes the following regions: 1, the construct containing full-length *TFAM* promoter, which is the natural promoter of *TFAM*; 2, the promoter construct with two discrete GCGC repeats (blue) and one NRF1 binding GCGCCTGCGC motif (orange); 3, the promoter construct only containing two discrete GCGC repeats; 4, the promoter construct with just the NRF1 binding GCGCCTGCGC motif. **(B)** The luciferase activities of wild-type NRF1 using the pGL4.20-*TFAM*-promoter constructs 1–4 from (A). **(C)** Western blot analysis of NRF1 wild-type and mutants in HEK293T cells. Δ96–176 and Δ177–284: DD and DBD deletion NRF1 mutants. **(D)** Luciferase activities driven by different NRF1 mutants and the pGL4.20-*TFAM*-promoter construct4 containing the intact GCGCCTGCGC motif as shown in (A). **(E)** Designed pGL4.20-*TFAM*-promoter constructs with different GCGCCTGCGC mutation motifs. The sequence in black at the top corresponds to a natural promoter, and the other constructs below are its mutants for luciferase activity. Mut1-9, differently mutated pGL4.20-*TFAM*-promoter constructs based on the construct4, and the mutated nucleotides are shown in lower case and colored blue. **(F)** Luciferase activities driven by different mutated pGL4.20-*TFAM*-promoter constructs from (E) and wild-type NRF1. Data were the averages of the measurements with the standard deviation (SD). ***P < 0.001.

Given that NRF1 DBD alone can bind to the single TGCGC repeat DNA, we asked if the NRF1 monomer or the single TGCGC repeat-containing promoter is sufficient for the transcriptional activity of NRF1. Previously, NRF1 has been reported to bind to the *TFAM* promoter and activate its transcription (30). We further analyzed the human *TFAM* promoter and found that its proximal promoter region contains two discrete GCGC motifs and an intact GCGCCTGCGC motif (Figure 5A). Thus, we took the *TFAM* promoter as an example to analyze the transcriptional activity of NRF1. We first generated four pGL4.20-*TFAM*-promoter constructs containing either two discrete GCGC motifs, the intact GCGCCTGCGC motif, both, or the full-length *TFAM* promoter and conducted luciferase reporter assays with NRF1 (Figure 5A). Our luciferase activity results revealed that the *TFAM* promoter region containing the intact GCGCCTGCGC motif exhibits a comparable transcriptional activity to the full-length *TFAM* promoter, while the promoter region containing two discrete GCGC motifs exhibits significantly lower transcriptional activity (Figure 5B). Furthermore, we explored the transcriptional activity of the NRF1 truncation mutants with DD and DBD individually deleted using the pGL4.20-*TFAM*-promoter construct containing the intact GCGCCTGCGC motif. We observed that both NRF1 truncation mutants display similar expression levels as the NRF1 full-length in HEK293T cells (Figure 5C), however, the transcriptional activity of both NRF1 mutants is reduced by ∼3-fold compared to the NRF1 WT (Figure 5D).

To examine the significance of the specific DNA sequence recognition by NRF1 in transcriptional activation, we evaluated the transcriptional activity of the NRF1 base-interacting residue mutants and pGL4.20-*TFAM*-promoter constructs containing different GCGCCTGCGC mutation motifs (Figure 5E). Despite the NRF1 WT and mutants displaying similar expression levels (Figure 5C), all NRF1 mutants exhibit ∼3-fold decreased transcriptional activity compared to the NRF1 WT (Figure 5D). Additionally, we observed that replacing any nucleotide of the GCGC repeat in the GCGCCTGCGC motif results in a ∼3-fold decrease in the transcriptional activity of NRF1 (Figure 5F). Simultaneous mutation of any two palindromic nucleotides in the two GCGC repeats, or substituting one GCGC repeat with atta in the GCGCCTGCGC motif, leads to a ∼6-fold decreased transcriptional activity compared to the wild-type *TFAM* promoter (Figure 5F). All of these luciferase activity data are in line with our ITC binding data for the NRF1 mutants and GCGCATGCGC DNA variants (Figure 3D, Table 2, Supplementary Figure S3). Taken together, our luciferase reporter assays indicate that both the NRF1 dimerization and the intact consensus sequence GCGCNTGCGC are essential for the effective transcriptional activation of the NRF1 target genes, such as *TFAM*, while the NRF1 DBD alone or the single TGCGC repeat-containing promoter is insufficient for the transcriptional activity of NRF1.

## Discussion

NRF1 plays significant roles in various cellular functions, including both mitochondrial and non-mitochondrial processes. Analysis of ChIP-Seq datasets shows that the ChIP-Seq identified NRF1 target genes in SK-N-SH cells are involved in various cellular processes such as mitochondrial function, RNA metabolism, DNA damage repair, cell cycle regulation, protein translation initiation, and ubiquitin-mediated protein degradation (42). Aberrant NRF1 has been linked to tumorigenesis, as some key oncogenes are regulated by NRF1, including genes related to glioblastoma (43), breast tumorigenesis (44,45), bladder cancer (46), prostate cancer (47), liver hepatocellular carcinoma (48) and EBV-associated gastric cancer (49). Therefore, in addition to being a crucial transcription factor, NRF1 has also emerged as a valuable biomarker for cancer diagnosis and prognosis. So far, the functions and potential pathogenesis involving abnormal expression and mutations of NRF1 have been widely studied, however, the molecular mechanisms underlying its role in specific promoter binding and gene regulation remain unclear.

Previous studies have reported that the N-terminal region (aa 1–78) of NRF1 mediates its dimerization, followed by a nuclear localization signaling motif (aa 88–116) and a DNA-binding region (aa 109–305) (5,50). Our binding and structural analyses reveal that the fragment covering the residues 54–172 serves as the dimerization domain (DD), the following fragment covering the residues 201–284 functions as the DNA-specific binding domain of NRF1, and the two domains are connected through a flexible linker1 (Figure 2A, Supplementary Figure S4C). In addition, we found that the DD not only mediates the homodimerization of NRF1, but also contributes to the non-sequence specific DNA binding of NRF1 (Figure 2D and 3E, Supplementary Figure S5C and S5D). When binds with DNAs, only one NRF1 monomer adopts a structural conformation that is close to the AlphaFold2 predicted model, and the DBD and DD of the other NRF1 monomer adopts a U-turn conformation, resulting in the two DBDs of the NRF1 homodimer bind to the consensus sequence GCGCNTGCGC from nearly the opposite side of the DNA duplex (Figure 2A and B). Thus, both DD and DBD of NRF1, along with the flexible linker1 between them, cooperatively contribute to the recognition of the GCGCATGCGC or GCGCATGCGC-like sequences in the promoters. Notably, zebrafish NRF1, sea urchin P3A2, and Drosophila EWG (erect wing gene product), which are homologs of human NRF1, have been identified as important regulators involved in the expression of genes that are vital for various developmental processes (4,51,52). Sequence alignment shows significant similarity between these homologs and human NRF1 over the DD and DBD (Supplementary Figure S4C), implying that they may bind to DNA in a similar mode.

Structural comparison of the NRF1 DBD–dsDNA and NRF1 dimer–dsDNA complexes shows that the arrangement of the two DBD molecules in the DBD-dsDNA complex is different from that of the DBDs in the NRF1 dimer–dsDNA complex (Figure 4D). We speculated that the different arrangement of DBD should be due to the presence of DD in the NRF1 dimer, that is DD restricts the orientation of the DBD in the NRF1 dimer-dsDNA complex, enabling the two DBDs to bind to an intact GCGCATGCGC motif (Figure 2A, B and 4D). In addition, our ITC and luciferase reporter assay results showed that the NRF1 proteins that lack DD or contain DBD mutations exhibit reduced binding affinity and significantly lower transcriptional activity compared to the NRF1 dimer (Figure 3D, 4A and 5D). Similarly, mutating any nucleotide of the GCGC repeat in the GCGCNTGCGC motif also significantly reduces the binding affinity and transcriptional activity of NRF1 (Figure 5F, Table 2). These findings are consistent with previous studies demonstrating that the NRF1 (aa 88–144) deletion mutant impairs its nuclear localization and transcriptional activity (50), and mutations of the GCGC sequence of the NRF1 binding motif in target gene promoters also decrease NRF1 transcriptional activity (16–18,53). Therefore, these results emphasize that the cooperativity between the DBD and DD is essential for the promoter recognition and transcriptional activity of NRF1.

Phosphorylation is a well-studied post-translational modification of NRF1 that has been shown to activate NRF1 by enhancing its DNA binding ability or promoting NRF1 dimerization. For example, the casein kinase II-mediated phosphorylation on the N-terminus of NRF1 (5) and the AKT-mediated phosphorylation of T109 of NRF1 were reported to enhance NRF1 transcriptional activity by increasing its DNA binding ability (30). In addition, ATM-mediated phosphorylation of T259 has been implicated in triggering the dimerization of NRF1 (54). However, our structural analysis reveals that residues T109 and T259 are located in the DD and DBD regions of NRF1, respectively, and both residues form hydrogen bonds with the DNA backbone (Figure 3C). When T109 or T259 is mutated to mimic phosphorylation by substituting them with aspartic acid (T109D or T259D), steric clashes and electrostatic repulsion would occur between the side chain of T109D or T259D and the DNA backbone. This observation was further supported by our ITC experiment that the T109D mutant reduces the binding affinity by ∼5-fold compared to the wild-type NRF1 (Supplementary Figure S7). Thus, our complex structure indicates that the phosphorylation of T109 and T259 might not increase the DNA binding ability or promote the dimerization of NRF1. The phosphorylation-related activation for NRF1 needs to be further studied.

Although the NRF1 DD shares a modest 16% sequence identity with transcription factor SRF over its MADS domain, our structure search shows that the NRF1 DD exhibits the closest structural homolog to the SRF MADS domain. The SRF MADS domain consists of two helices α1 and α2, along with two antiparallel β-strands β1 and β2. Structural comparison of the NRF1 DD and the SRF1 MADS reveals that similar to the NRF1 DD, the SRF MADS domain dimerizes through forming an intermolecular 4-stranded β-sheet, which is sandwiched by two antiparallel helices on one side and two short helices on the other side (Supplementary Figure S5B, S10A and S10B), suggesting that the NRF1 DD and the SRF MADS exhibit a similar dimerization interface. However, in the SRF MADS–dsDNA complex, SRF MADS binds to dsDNA with two amphipathic α-helices inserted into the DNA major grooves, and its extended N-terminal loops contacting the DNA minor grooves (Supplementary Figure S10B). This binding mode not only determines its DNA binding specificity but also induces DNA bending (36–38). In contrast, our NRF1 dimer-dsDNA structure reveals that only one NRF1 DD binds to the backbone of the DNA duplex, while the DBD determines the sequence-specific binding of NRF1 (Figure 2A, Supplementary Figure S5C and S10A). Apart from the dimerization and DNA binding, the SRF1 MADS domain also modulates the interaction with its partners, enabling SRF and its partners to bind to different sequences within promoters. For example, in the SAP-1–SRF–dsDNA ternary complex, the SAP-1 B-box interacts with the SRF MADS domain, and the SAP-1 ETS domain and the SRF MADS domain cooperatively bind to the EBS site and CArG box in the promoter (Supplementary Figure S10C) (37,55). Although this binding mode is similar to that observed in the NRF1 dimer-dsDNA complex, the phylogenetic analysis reveals that NRF1 has no apparent evolutionary relationship with SRF.

NRF2 (also called GABP) was named after NRF1 because they both function in regulating the expression of nuclear-encoded mitochondrial proteins (56). However, NRF2 functions as a multi-subunit transcriptional activator, comprising a DNA-binding subunit α, known as GA binding protein α (GABPα), and four co-activator subunits β1, β2, γ1, and γ2 that form heteromeric complexes with GABPα being responsible for DNA binding (57). GABPα possesses an ETS (Erythroblast Transformation Specific) DNA binding domain that specifically recognizes the core GGAA motif (56,58). The molecular basis of GABPα-GABPβ1 heterodimer binding to the core GGAA motif has been studied in mice (Supplementary Figure S11) (59). Structural analysis shows that NRF1 and NRF2 exhibit distinct structural features and diverse binding modes to their respective binding motifs. Therefore, our findings emphasize the significance of NRF1’s distinct structure and DNA binding mode in mediating its transcriptional functions.

## Supplementary Material

gkad1162_supplemental_file

## Data Availability

The crystal structures of the NRF1 homodimer and DBD in complex with dsDNA have been deposited in the Protein Data Bank with accession codes 8K4L and 8K3D, respectively.
