# Supplementary material for: Molecular mechanism of specific DNA sequence recognition by NRF1
Source: Nucleic Acids Res. 2023 Dec 6;52(2):953–66. doi: 10.1093/nar/gkad1162 (PMC10810270; doi:10.1093/nar/gkad1162)
Supplement: gkad1162_supplemental_file [file gkad1162_supplemental_file.pdf]

SUPPLEMENTARY INFORMATION FOR
Molecular mechanism of specific DNA sequence recognition by NRF1

Ke Liu[#,*], Weifang Li[#], Yuqing Xiao, Ming Lei, Ming Zhang, and Jinrong Min[*]
Hubei Key Laboratory of Genetic Regulation and Integrative Biology, School of Life Sciences, Central China Normal University, Wuhan 430079, PR China
[#] These authors contributed equally to this work.
[*] To whom correspondence should be addressed. Email: keliu2015@ccnu.edu.cn or minjinrong@ccnu.edu.cn.

Table S1. Data collection and refinement statistics

Structure	NRF1 (aa 177-284)<br>5'-GGTGCGCATGCGCACC-3'<br>3'-CCACGCGTACGCGTGG-5'	NRF1 (aa 54-284)<br>5'-ATGCGCATGCGCAT-3'<br>3'-TACGCGTACGCGTA-5'
**PDB ID**	8K3D	8K4L
**Data Collection**		
Space group	$C222_1$	$P2_12_12_1$
Cell dimensions		
a,b,c [Å]	63.84, 64.71, 98.52	37.76, 118.83, 121.82
α, β, γ [°]	90, 90, 90	90, 90, 90
Resolution [Å]	23.05-2.30 (2.38-2.30)[a]	42.53-2.10 (2.16-2.10)
Completeness [%]	97.3 (99.5)	100.0 (100.0)
R_{merge}[b]	0.080 (0.331)	0.072 (0.568)
I/σI	7.0 (1.6)	18.2 (4.4)
CC1/2	0.993 (0.874)	0.978 (0.955)
Redundancy	2.9 (2.8)	12.3 (10.3)
**Refinement**		
Resolution (Å)	23.05-2.30	38.43-2.10
Reflections (working set)	9013	32910
Reflections (test set)	429	1598
No. atoms/B-factor ($Å^2$)	1280/43.31	4015/48.70
Protein	859/45.46	1570/50.50
DNA	325/38.34	284/38.38
Water	96/40.87	307/47.56
R_{work}/R_{free}[c]	0.194/0.239	0.193/0.243
RMS deviations		
Bond lengths (Å)	0.009	0.009
Bond angles (°)	1.015	1.061
**Ramachandran Plot % residues**		
Favored	98.1%	97.9%
Allowed	1.9%	2.1%
Outliers	0	0

[a] Numbers in parentheses represent the highest resolution shell.

[b] Rmerge = ∑hkl∑i|Ii(hkl)−<I(hkl)>|/∑hkl∑iIi(hkl).

[c] R-factor = ∑hkl||Fo|−|Fc||/∑hkl|Fo|.

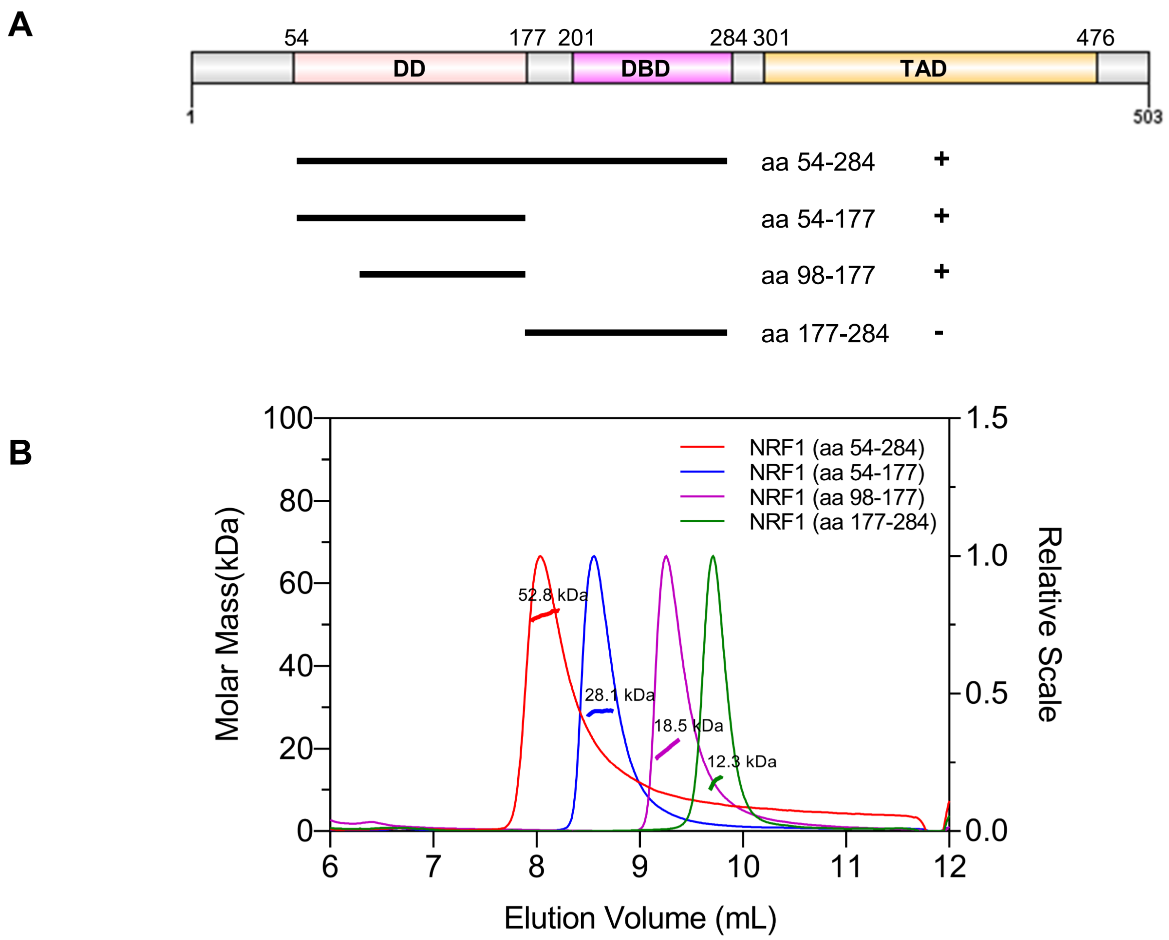

Figure S1. Dimerization analysis of human NRF1. **(A)** Schematic diagram of the NRF1 fragments used in the dimerization analysis. Domain architecture of human NRF1 is shown at the top of the panel. Dimerization domain (DD, pink), DNA binding domain (DBD, dark pink), and transactivation domain (TAD, yellow) are shown. **(B)** SEC-MALS analysis of the different deletion constructs depicted in (A). The estimated molecular weights of each sample based on the elution volumes are labeled.

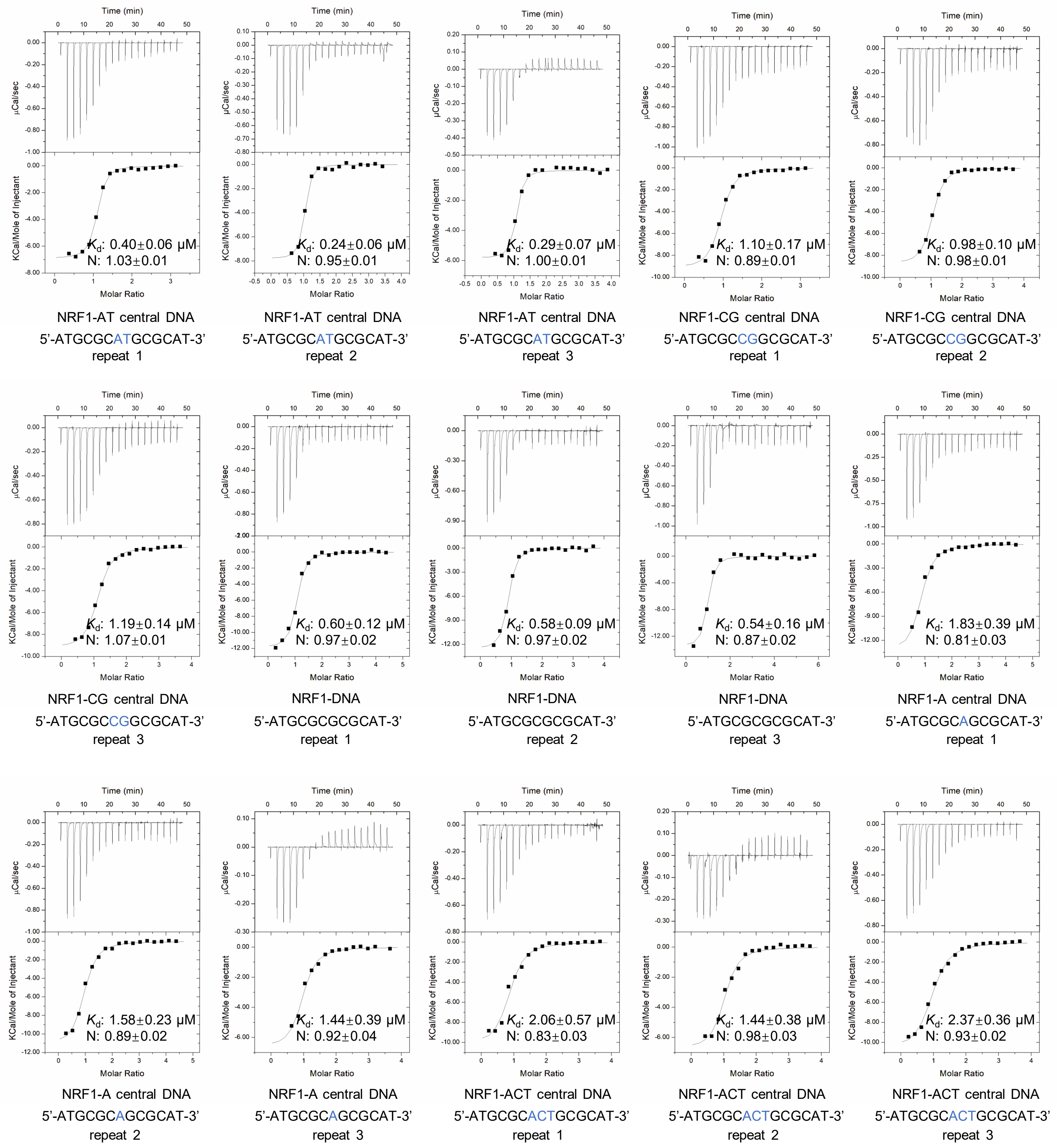

Figure S2. Binding affinities of NRF1 (aa 54-284) to dsDNAs containing different numbers of nucleotides as the central spacer between two GCGC half-sites. Only one strand of the DNA duplex is shown. The ITC assays are conducted in triplicate. The nucleotides of the central spacer are colored blue.

Continued

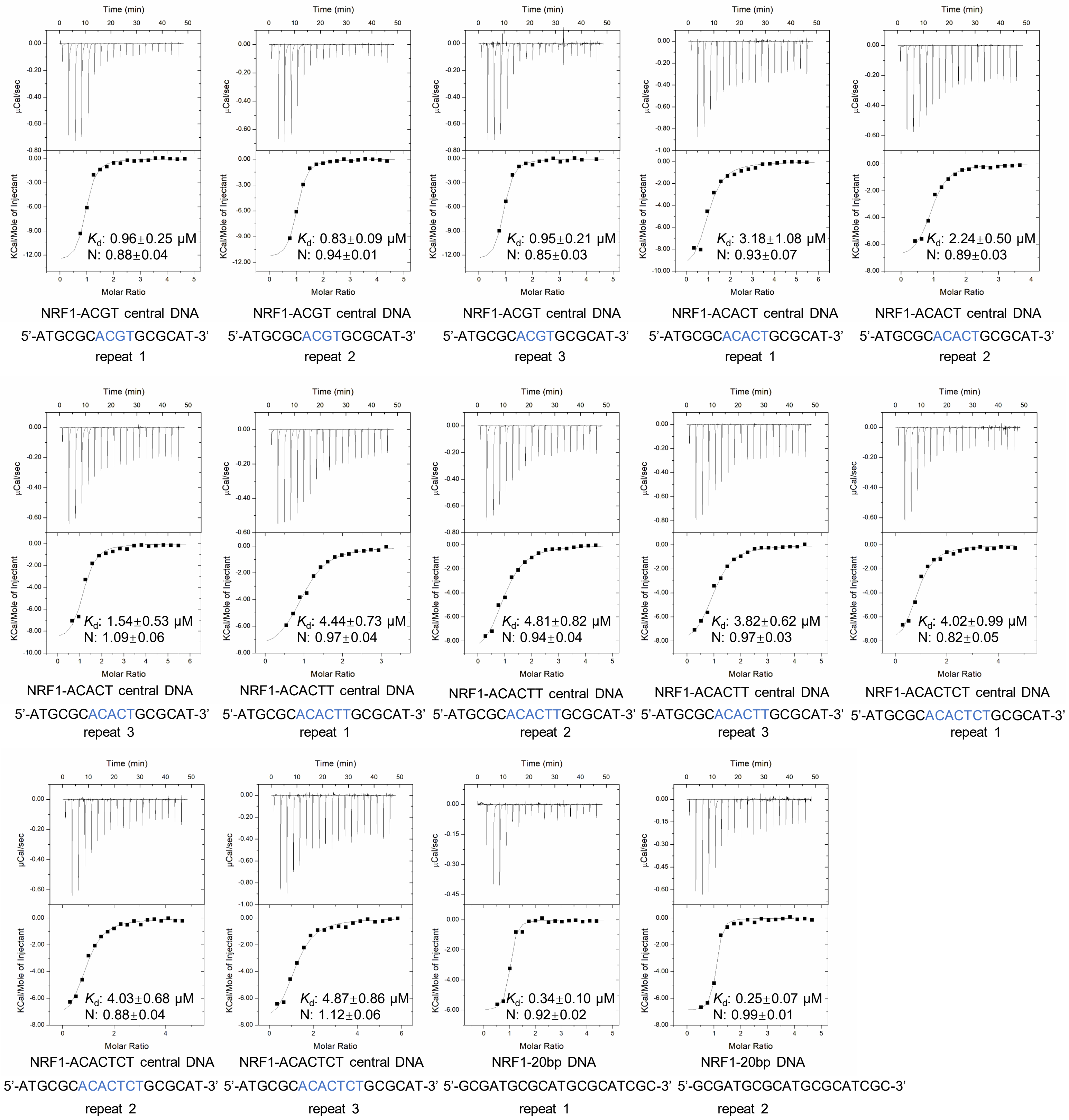

Figure S2. Binding affinities of NRF1 (aa 54-284) to dsDNAs containing different numbers of nucleotides as the central spacer between two GCGC half-sites. Only one strand of the DNA duplex is shown. The ITC assays are conducted in triplicate. The nucleotides of the central spacer are colored blue.

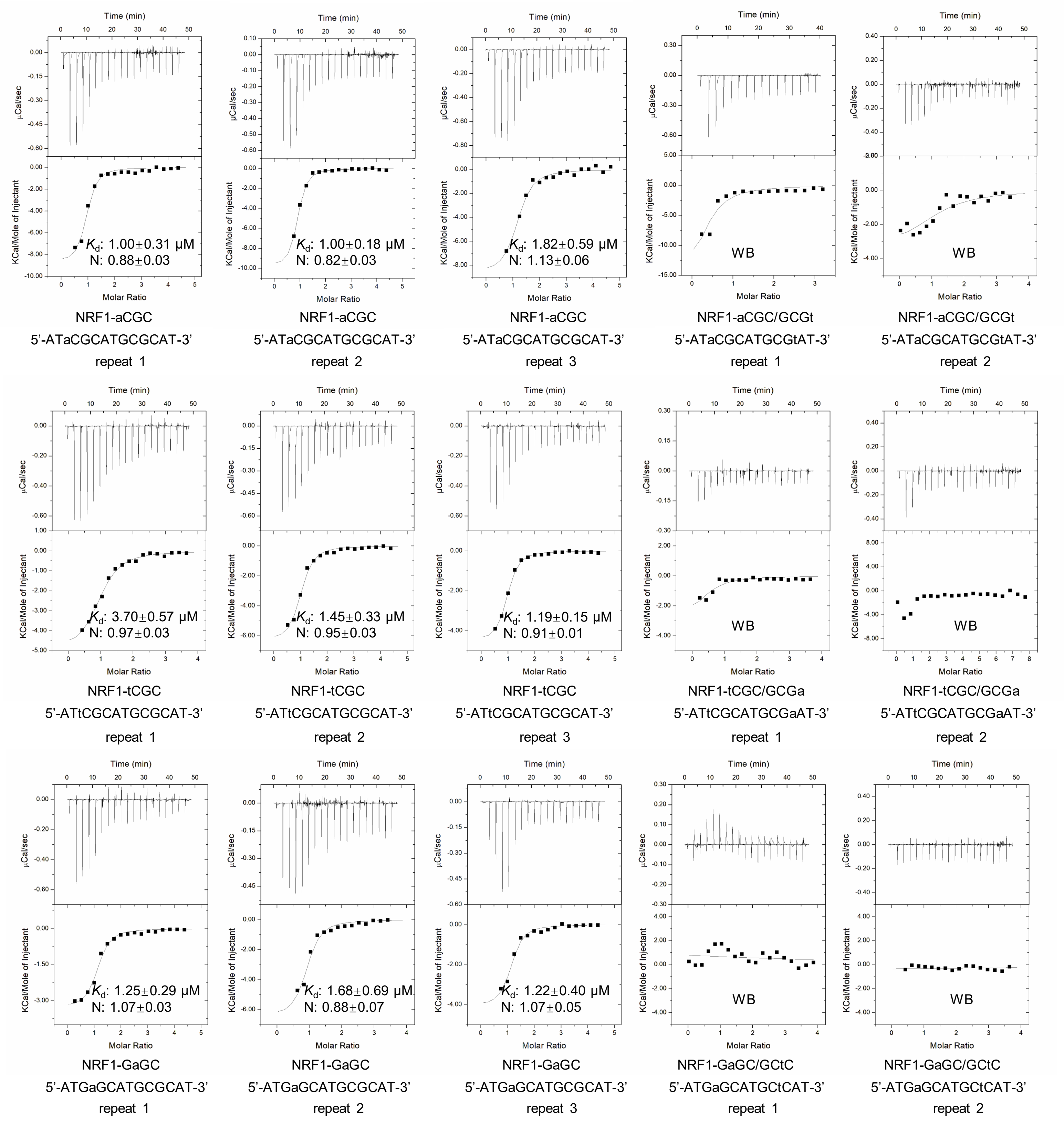

Figure S3. ITC curves of NRF1 (aa 54-284) binding to different dsDNA variants. Only one strand of the DNA duplex is shown. WB: weak binding.

Continued

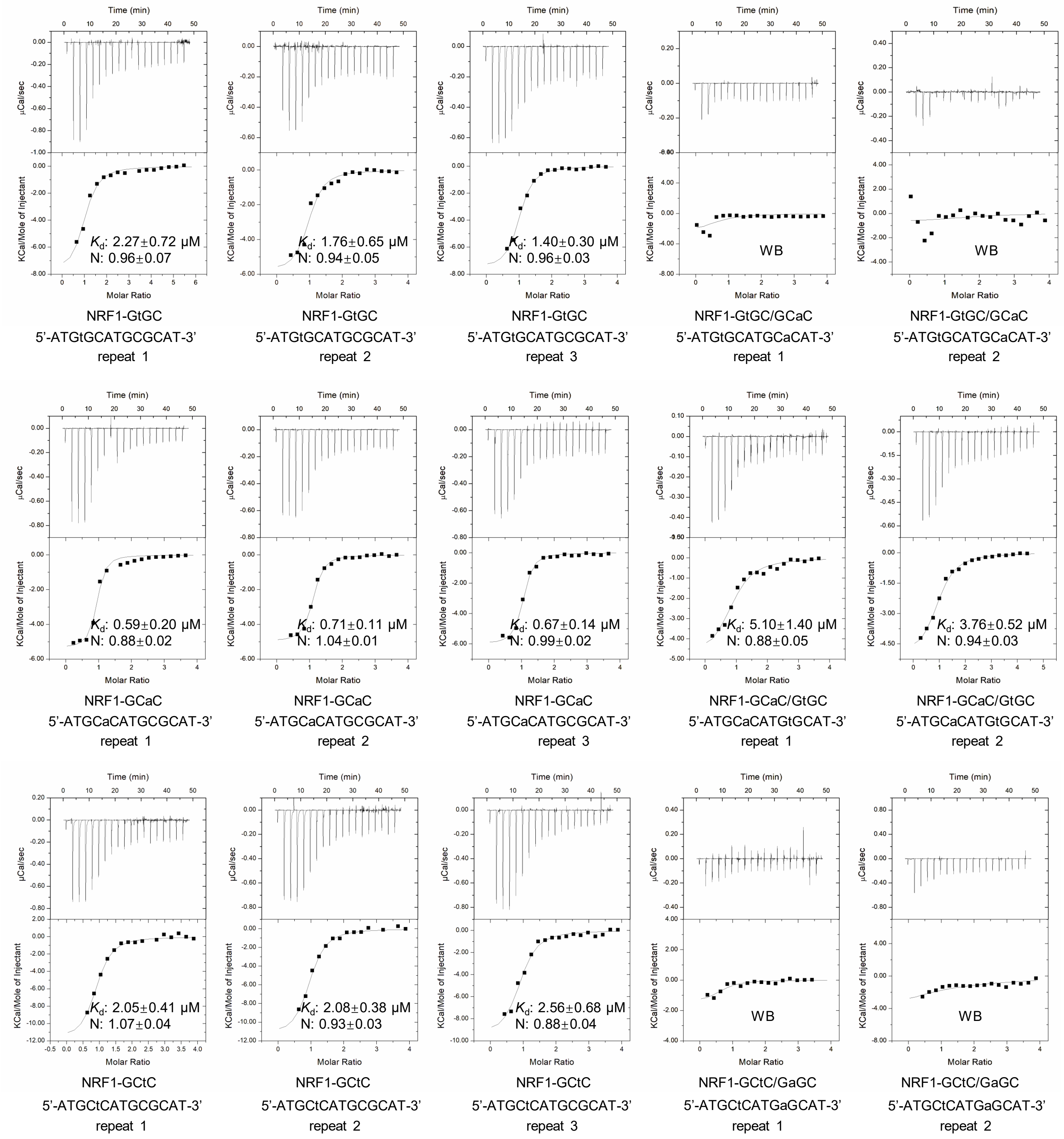

Figure S3. ITC curves of NRF1 (aa 54-284) binding to different dsDNA variants. Only one strand of the DNA duplex is shown. WB: weak binding.

Continued

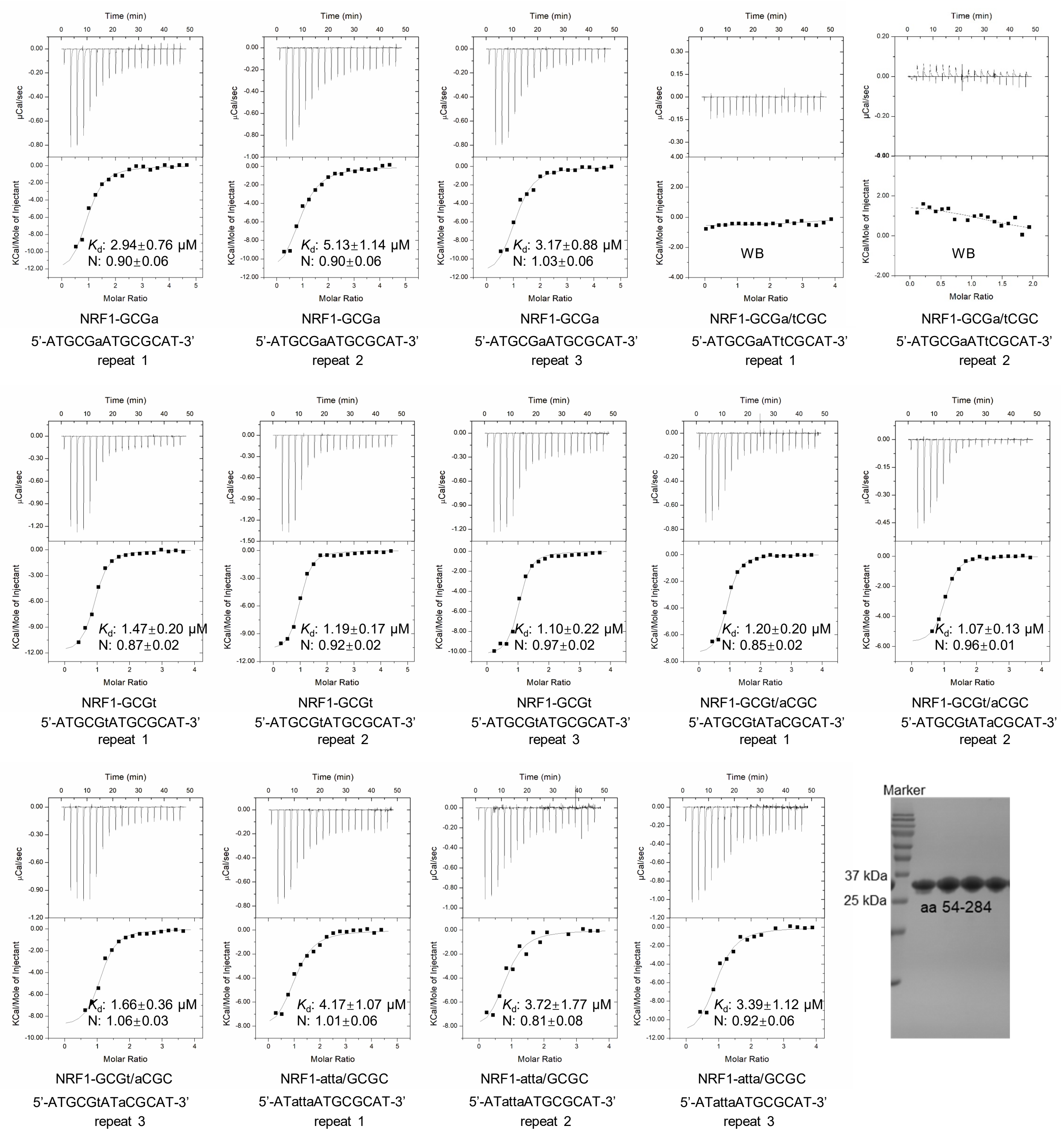

Figure S3. ITC curves of NRF1 (aa 54-284) binding to different dsDNA variants. Only one strand of the DNA duplex is shown. WB: weak binding. SDS-PAGE analysis of NRF1 (aa 54-284) sample used for the ITC assays in this Figure.

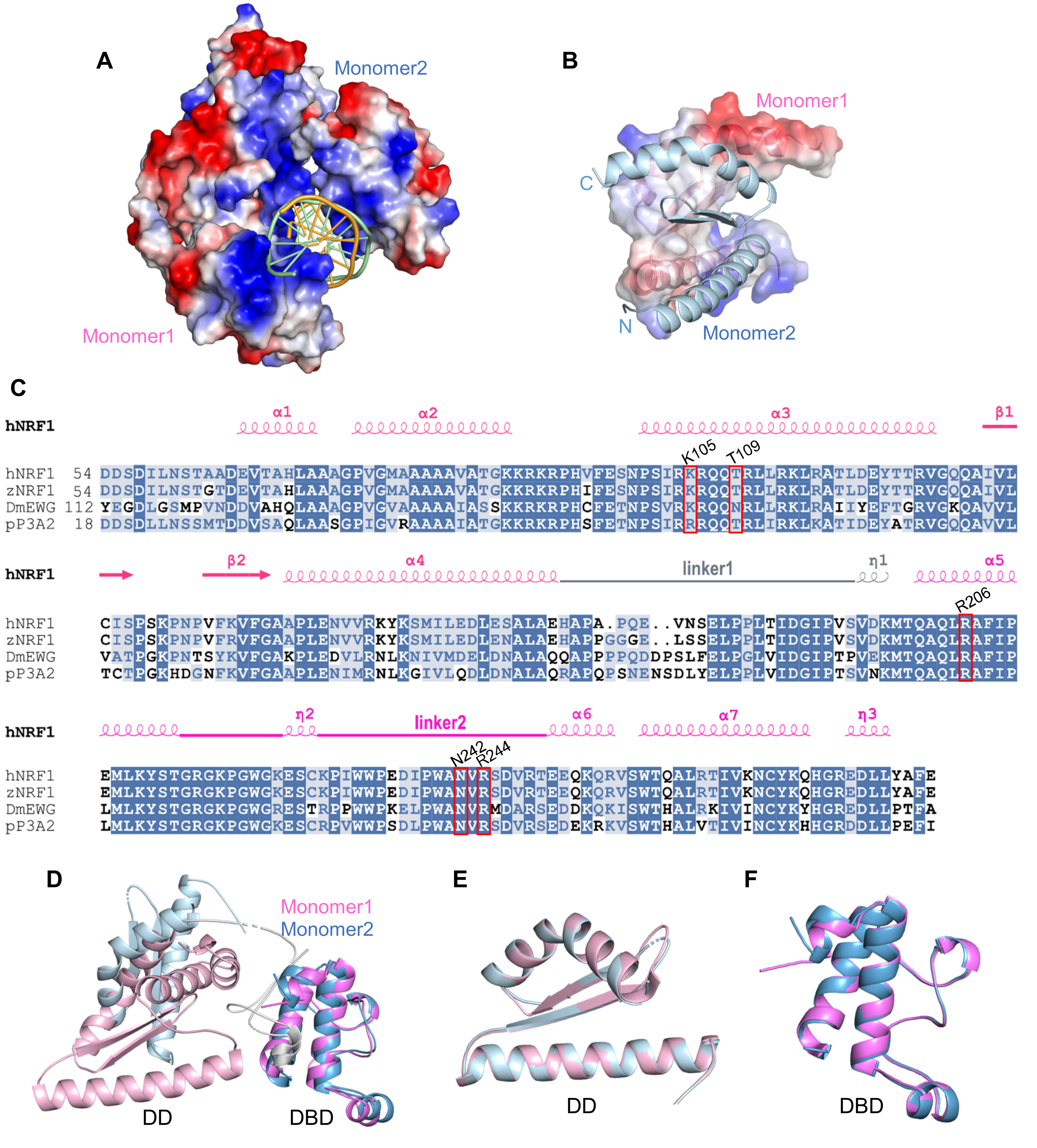

Figure S4. Crystal structure of the NRF1 homodimer in complex with the ATGCGCATGCGCAT dsDNA. (A) Complex structure of the NRF1 homodimer bound to ATGCGCATGCGCAT dsDNA in an electrostatic surface representation, which is related to Figure 2B. **(B)** Structure of the DD of NRF1. Two DDs are colored pink and blue, respectively, and one is only shown in a cartoon representation, and the other one is shown in a transparent electrostatic surface. **(C)** Sequence alignment of human NRF1 and its homologs from other representative species. hNRF1, human NRF1, uniport Q16656; zNRF1, Zebrafish NRF1, uniport Q90X44; DmEWG, Drosophila EWG, uniport Q24312; pP3A2, Purple sea urchin NRF1, uniport Q04073. The secondary structural elements of the DD and DBD are shown at the top of the sequences and colored pink and dark pink, respectively. The direct DNA backbone interacting residues of DD and base-specific binding residues of DBD are shown at the top of the sequence. The sequence alignment was generated using Clustalw2 and ESPript 3.0. **(D)** Superposition of two NRF1 monomers in the NRF1 dimer-dsDNA complex. The monomer1 and monomer2 are shown as cartoon representations in pink and blue with DBD colored in dark pink and deep blue, respectively. **(E)** and **(F)** Superposition of the DDs and DBDs of two NRF1 monomers. The DD and DBD are shown in the same way as Figure S4D.

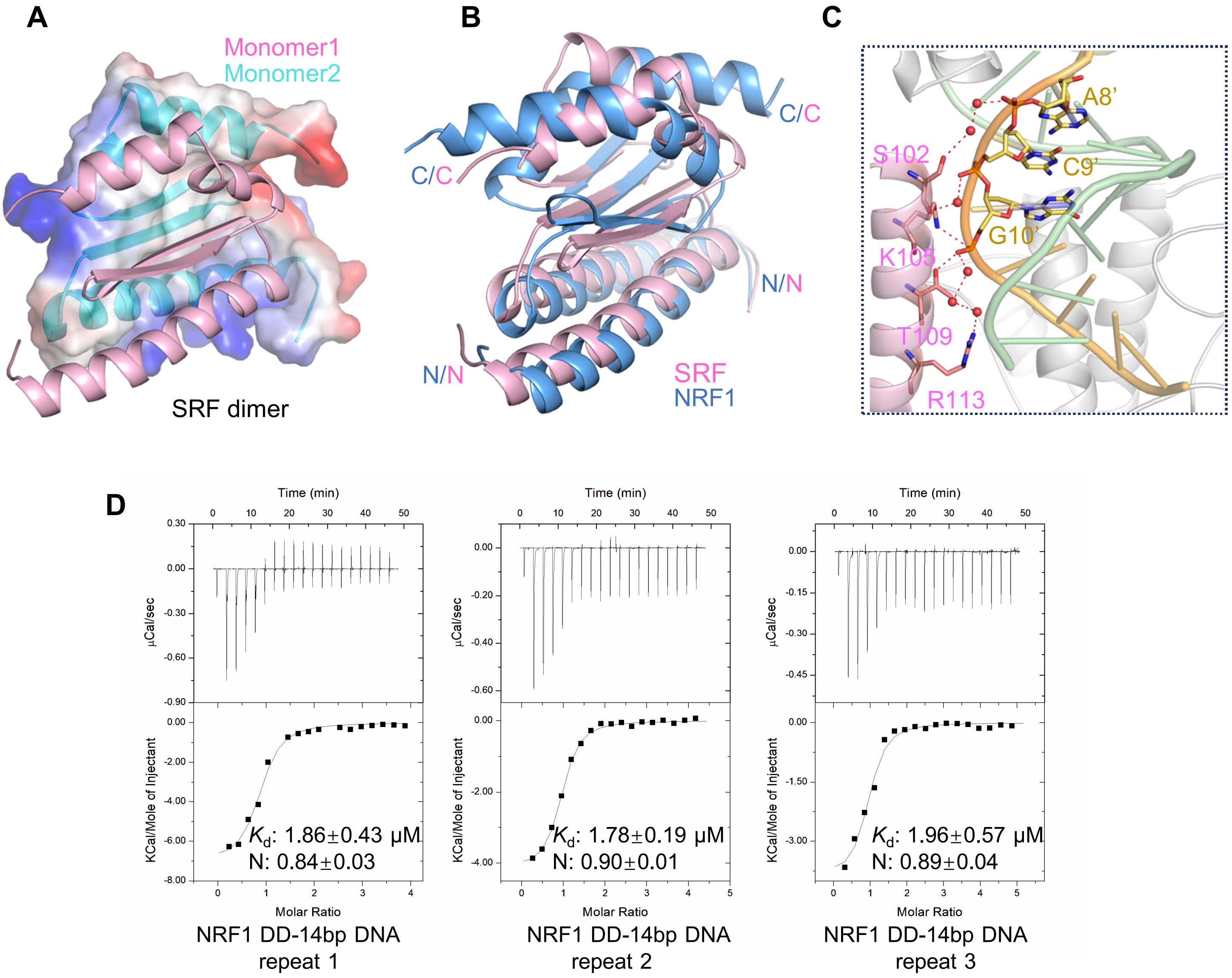

Figure S5. The NRF1 DD forms interactions with the ATGCGCATGCGCAT dsDNA. (A) The dimeric structure formed by SRF MADS domain (PDB: 1HBX)**. (B)** Structure alignment of SRF MADS dimer (pink) and NRF1 DD dimer (blue)**. (C)** The interactions between the NRF1 DD and the backbone of dsDNA. Hydrogen bonds formed between residues and dsDNA backbone are marked as red dashed lines**. (D)** ITC curves of the NRF1 DD (aa 54-177) binding to the ATGCGCATGCGCAT dsDNA.

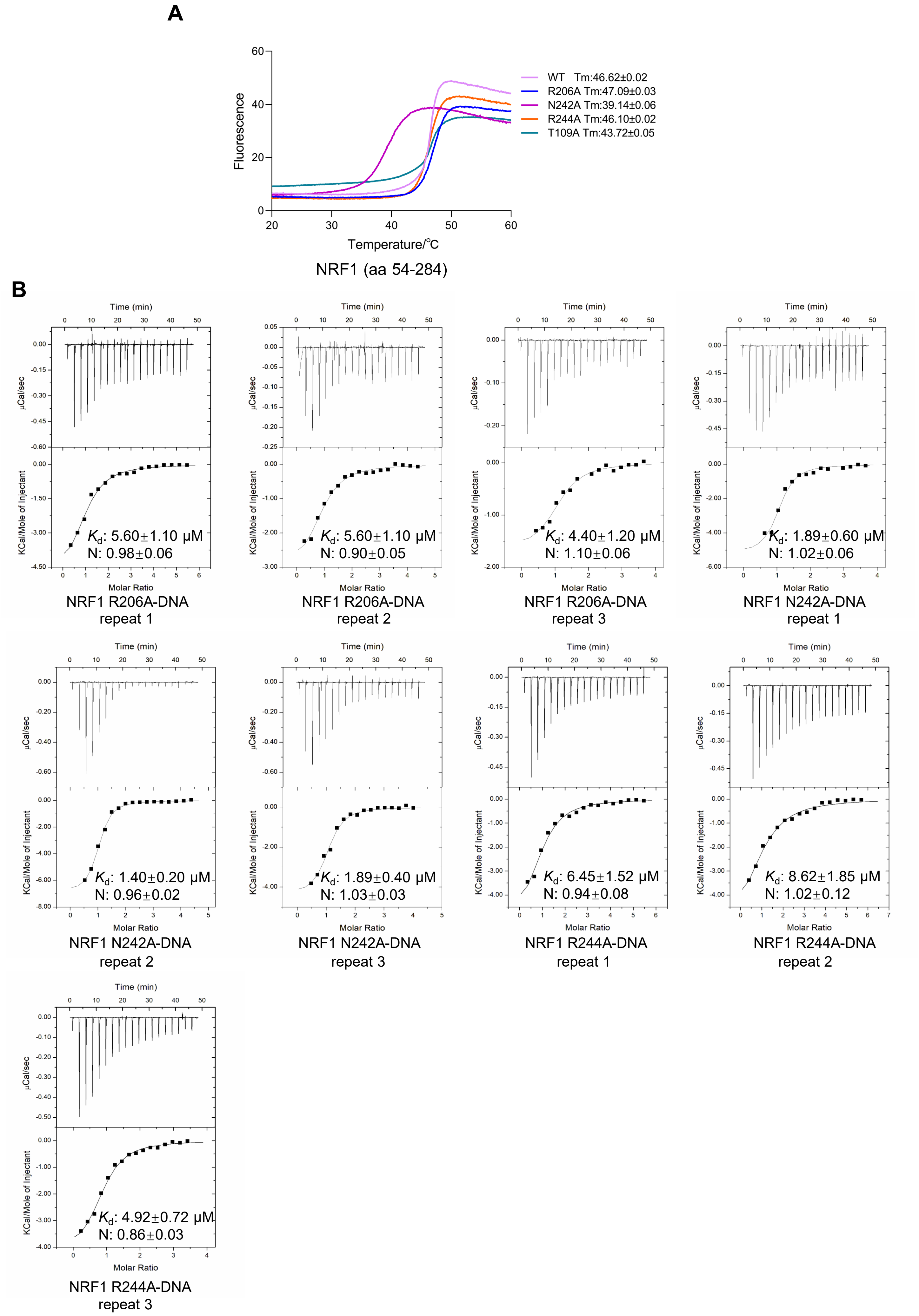

Figure S6. ITC binding curves of the NRF1 (aa 54-284) mutants to the ATGCGCATGCGCAT dsDNA. **(A)** The DSF analysis for the NRF1 (aa 54-284) mutants. **(B)** ITC binding curves of the NRF1 (aa 54-284) mutants to the ATGCGCATGCGCAT dsDNA.

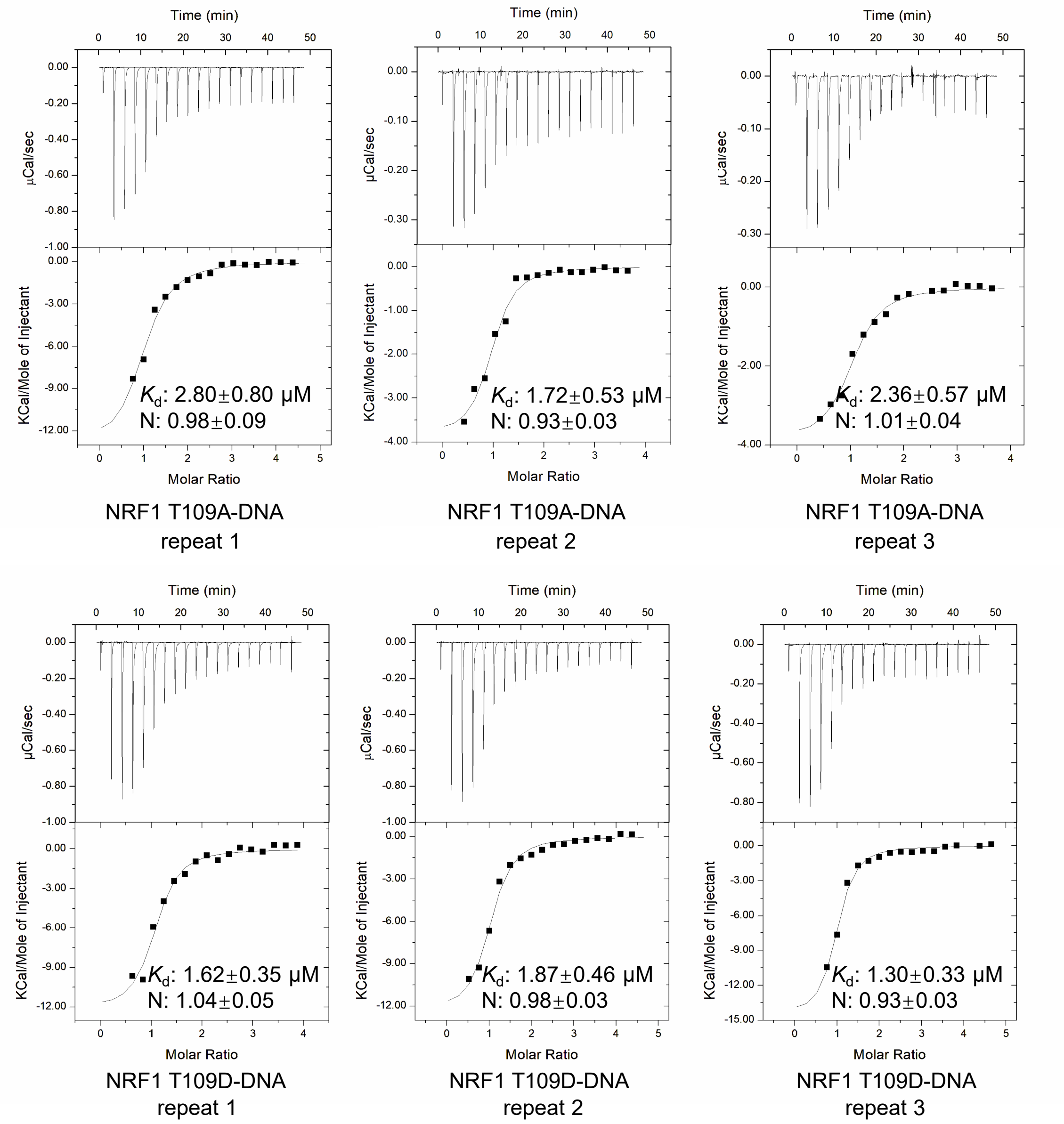

Figure S7. ITC binding curves of the NRF1 (aa 54-284) T109 mutants to the ATGCGCATGCGCAT dsDNA.

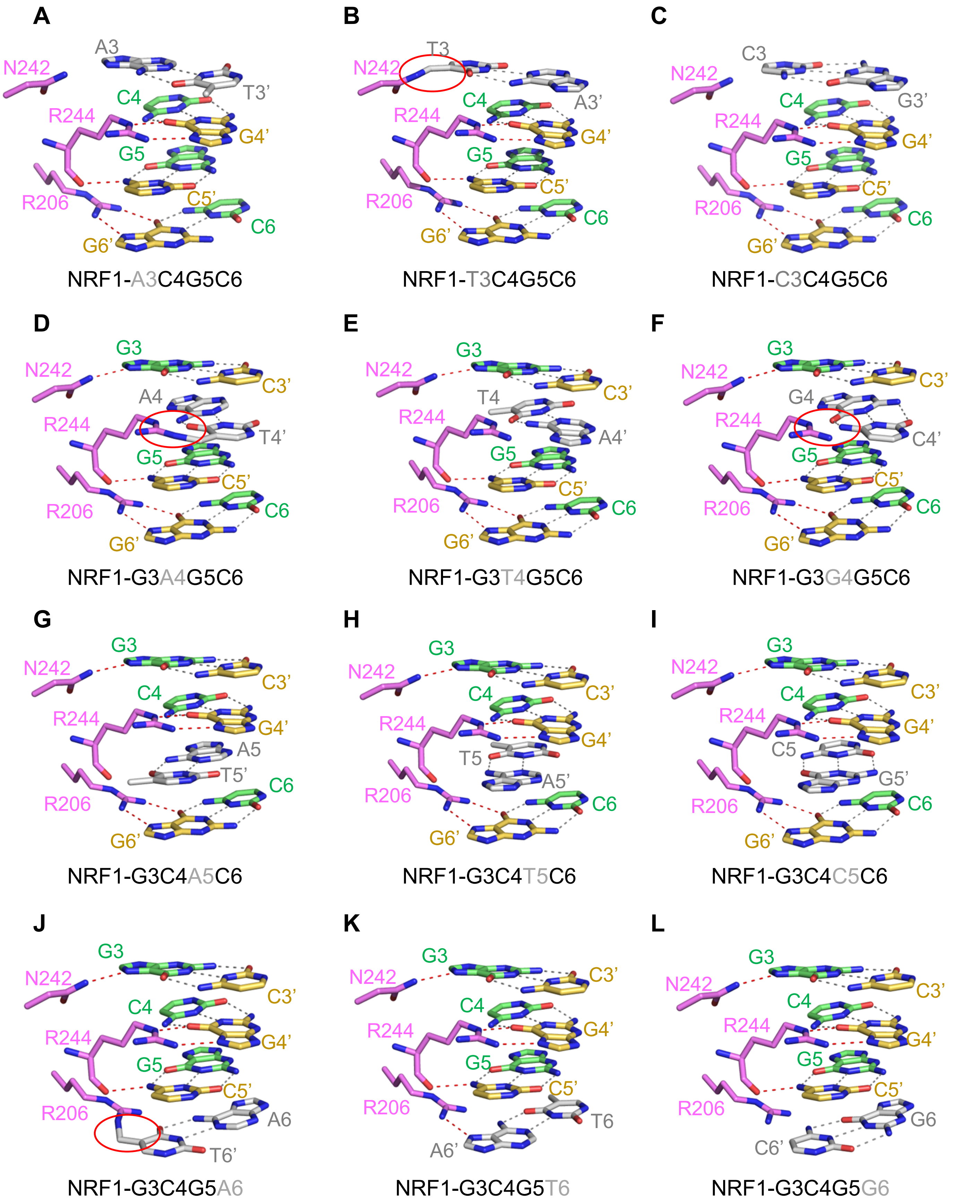

Figure S8. Structure models of NRF1 in complex with different dsDNA variants. The protein residues and DNA bases are shown in stick models, and the mutated nucleotides are colored gray. Hydrogen bonds formed between protein residues and DNA bases, and between DNA base pairs are marked as red and gray dashed lines, respectively. The red rings represent steric clashes.

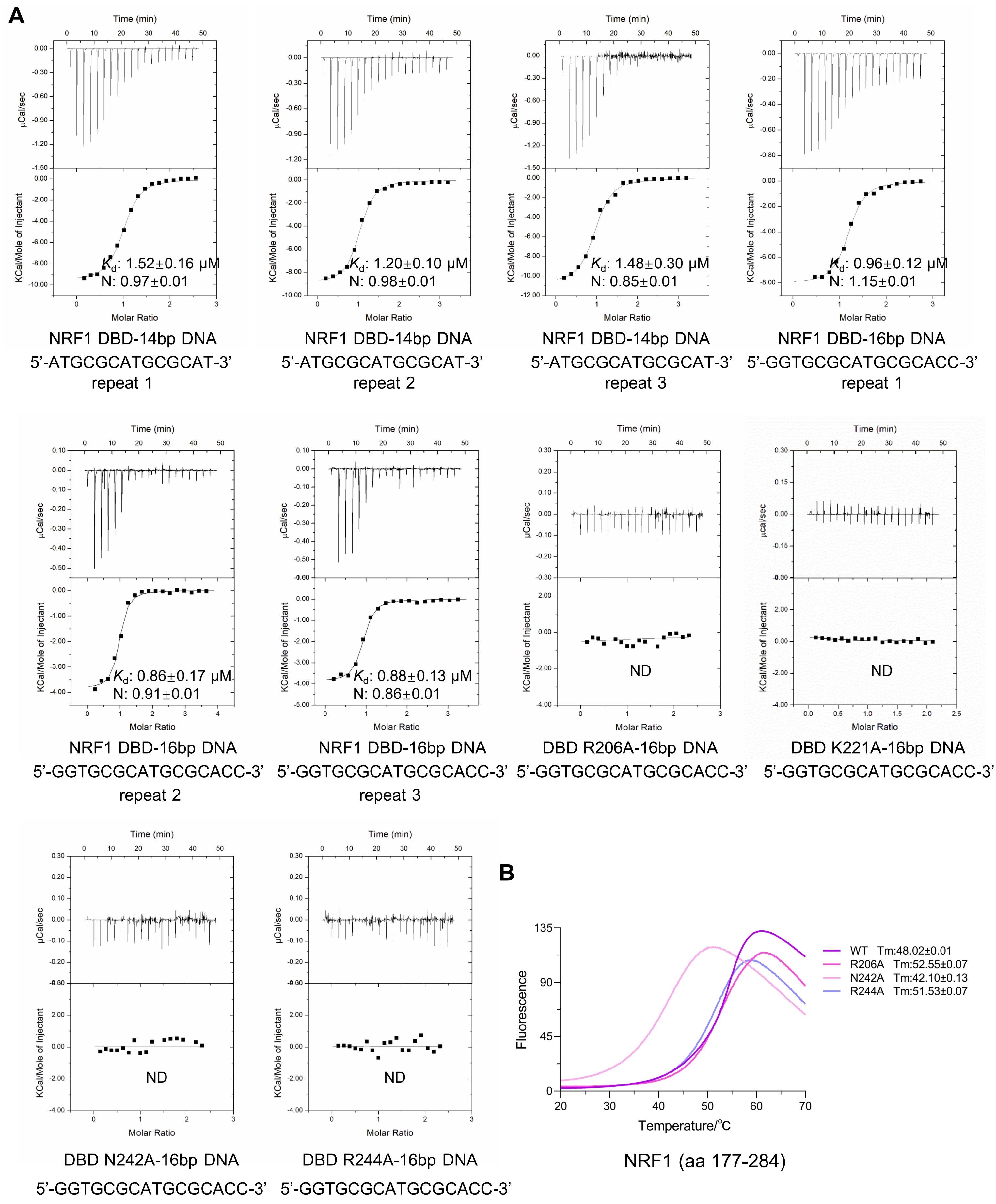

Figure S9. ITC binding curves of the NRF1 DBD (aa 177-284) and its mutants to 14bp and 16bp dsDNA. (A) ITC binding curves of the NRF1 DBD (aa 177-284) and its mutants to dsDNA. Only one strand of the DNA duplex is shown. ND: no detectable binding. **(B)** The DSF analysis for the NRF1 DBD alone and its mutants.

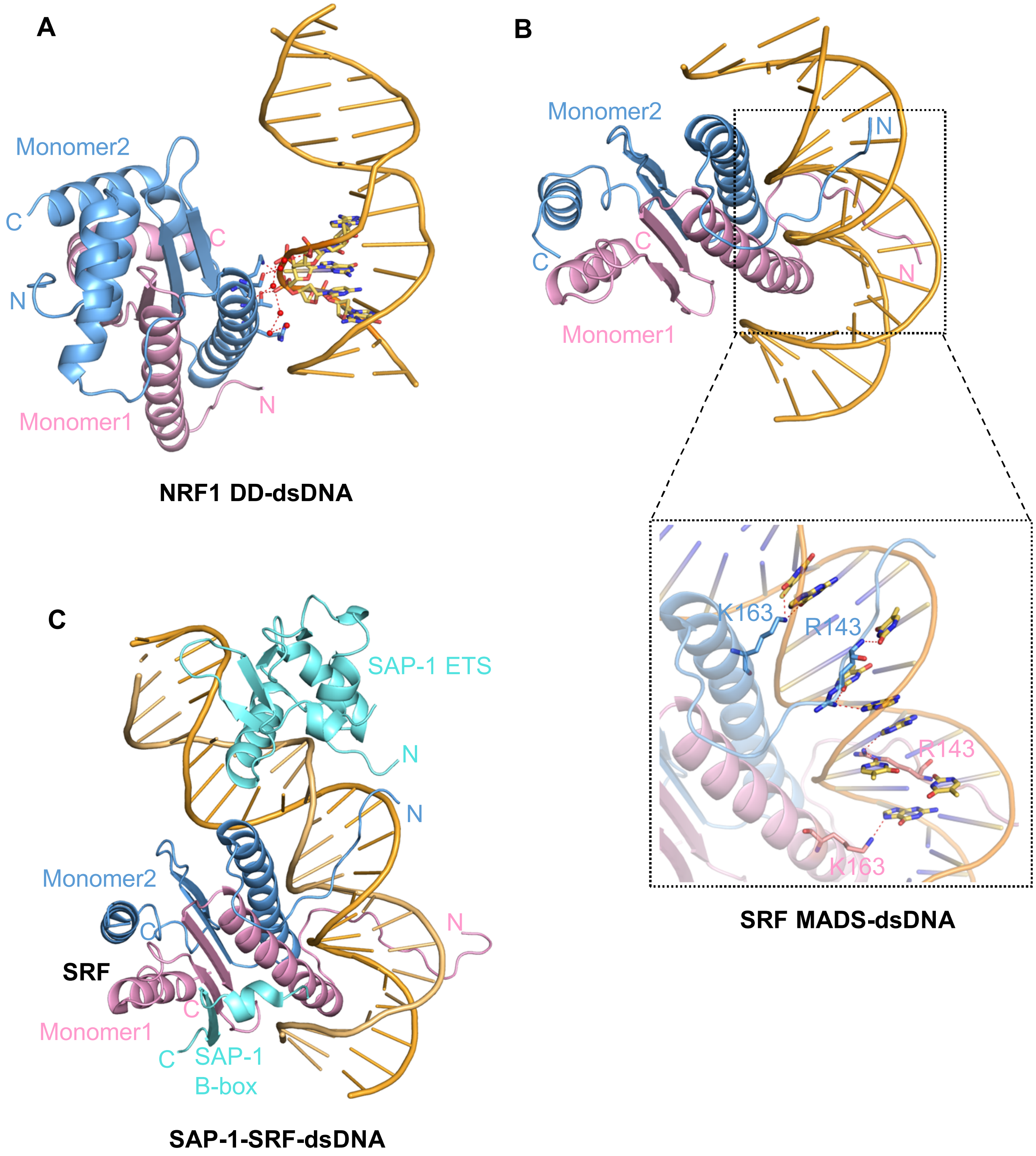

Figure S10. Structural comparisons of the NRF1 DD-dsDNA and SRF MADS-dsDNA complexes. **(A)** Complex structure of the NRF1 DD homodimer bound to dsDNA. The protein residues and DNA bases are shown in stick models. **(B)** Complex structure of the SRF MADS homodimer bound to dsDNA (PDB: 1SRS). The protein residues and DNA bases are shown in stick models, and detailed base interactions are also shown in the black zoom-in box. **(C)** Structure of the SAP-1-SRF complex bound to dsDNA (PDB: 1HBX). The SAP-1 binds to SFR via its B-box region. Hydrogen bonds formed between residues and dsDNA are marked as red dashed lines.

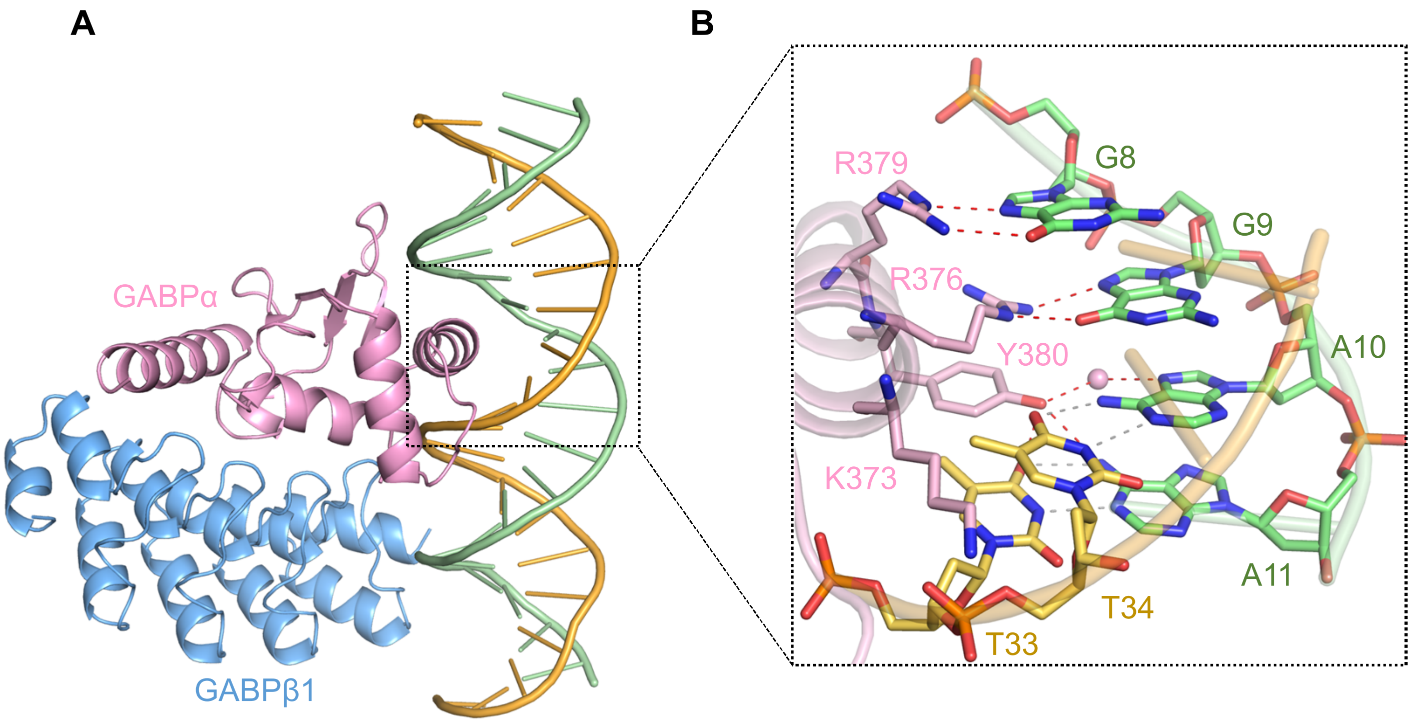

GABPα-GABPβ1 heterodimer-GGAA DNA

Figure S11. Complex structure of the GABPα-GABPβ1 heterodimer bound to the core GGAA motif dsDNA (PDB: 1AWC). (A) Overall structure of the GABPα-GABPβ1 heterodimer bound to the core GGAA motif dsDNA. **(B)** Detailed interactions between GABPα-GABPβ1 heterodimer and the GGAA sequence. Hydrogen bonds formed between residues and DNA bases are marked as red dashed lines, while DNA base interactions are marked as gray dashed lines.
